# Supplementary material for: ADF and Cofilin1 Control Actin Stress Fibers, Nuclear Integrity, and Cell Survival
Source: Cell Rep. 2015 Nov 19;13(9):1949–64. doi: 10.1016/j.celrep.2015.10.056 (PMC4678118; doi:10.1016/j.celrep.2015.10.056)
Supplement: Document S1. Supplemental Experimental Procedures, Figures S1–S7, and Table S1 [file mmc1.pdf]

SUPPLEMENTAL INFORMATION

SUPPLEMENTAL DATA

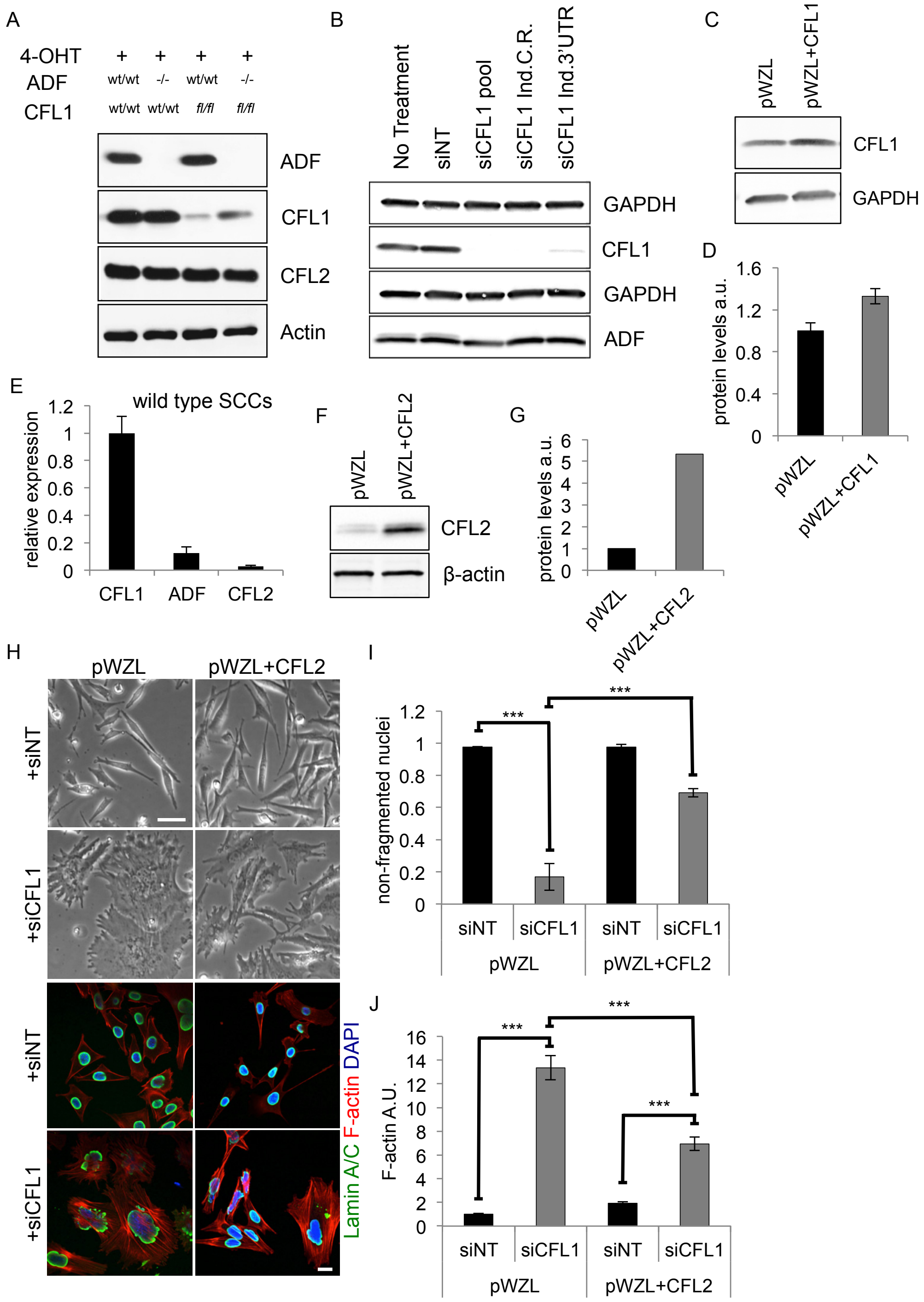

Figure S1. Depletion and re-expression of ADF/Cofilins (related to Figures 1, 2 and 5). (A) K14CreER^{T2} and K14CreER^{T2}/ADF$^{-/-}$/CFL1$^{wt/wt}$, K14CreER^{T2}/ADF$^{wt/wt}$/ CFL1$^{fl/fl}$ and K14CreER^{T2}/ADF$^{-/-}$/CFL1$^{fl/fl}$ mice were treated with 4-OHT. Proteins

were harvested from mouse tails and immunoblotting performed using anti-ADF, anti-CFL1, anti-CFL2 and anti-actin antibodies. **(B)** siRNA knock-down efficiency for CFL1 was tested by immunoblotting at 72h post-transfection of 32nM siRNA in wild type (wt) Squamous Cell Carcinoma cells (wt-SCCs). A pool of 4 siRNAs targeting CFL1 (siCFL1 pool) as well as individual siRNAs targeting the coding region (siCFL1 Ind.C.R.) or the untranslated (siCFL1 Ind.3'UTR) region of CFL1-mRNA were utilized. siNT represents non-targeting control siRNAs. GAPDH was used as a loading control. **(C)** Western blot comparing CFL1 levels in ADF-null SCCs infected with retroviruses containing an empty vector (pWZL) or a wt CFL1 expressing vector (pWZL+CFL1). **(D)** Quantification of the immunoblots using Image J based on the intensity of the protein species and normalized to the loading control (n=2, ± S.D.). **(E)** Relative expression of ADF, CFL1 and CFL2 in wt SCCs. Results were generated from two individual cell lines and data is presented ± S.D. The amplification efficiency of the primers was determined and taken into account for the data analysis after the results were normalized to an endogenous control (GAPDH). The primers used are provided in Table S1. **(F)** Western blot comparing CFL2 levels in ADF-null SCCs infected with retroviruses containing an empty vector (pWZL) or a wt CFL2 expressing vector (pWZL+CFL2). **(G)** Quantification of the immunoblots in (F) performed as described in (D). **(H)** Phase contrast images of ADF-null SCCs infected with an empty (pWZL) or CFL2 expressing vector (pWZL+CFL2) and treated with siNT or siCFL1 (top panels). Cells were stained for F-actin (red) and DAPI (blue) (bottom panels). Scale bars 60µm for phase and 20µm for IF images, respectively. **(I)** Quantification of pWZL or pWZL+CFL2 containing cells that displayed non-fragmented nuclei after siRNA treatment. Results presented as mean ± SEM (n=4). >100 cells were counted per condition per repeat. *** One-way ANOVA with Tukey's multiple comparison test, p-value ≤ 0.0001. **(J)** Quantification of F-actin levels of the same cells as in (I). ≥69 cells quantified per condition (n=3). Data presented as mean ± SEM. *** One-way ANOVA with Tukey's multiple comparison test, p-value ≤ 0.0001.

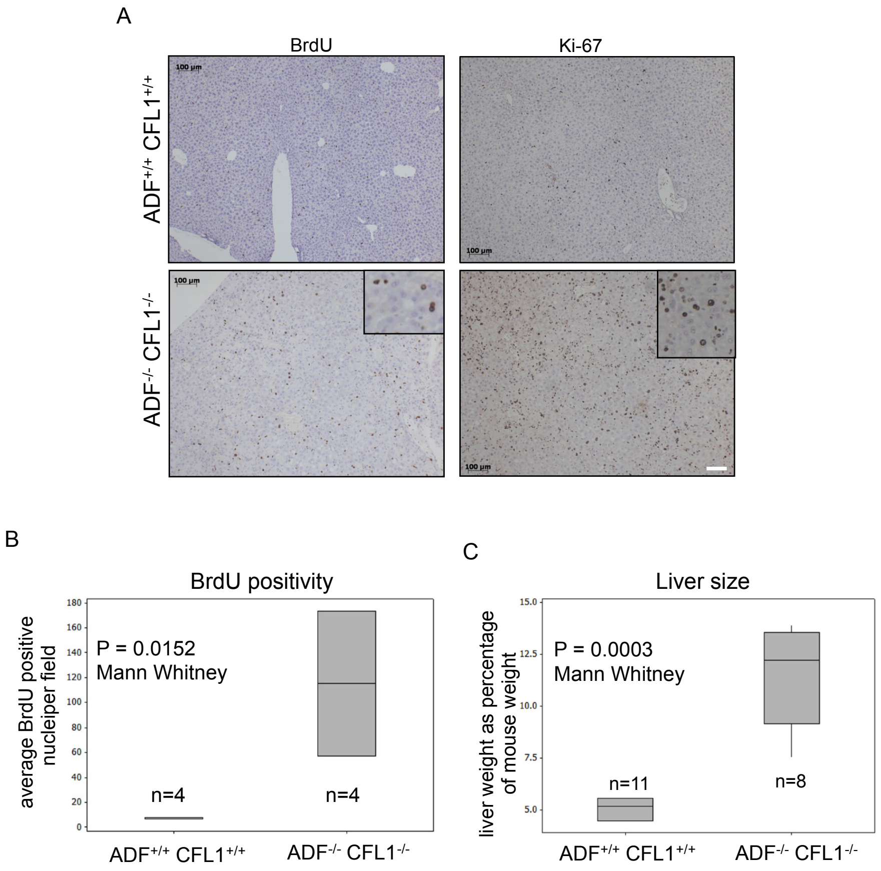

Figure S2. ADF and CFL1 gene deletion *in vivo* triggers hepatocyte hyperproliferation and liver enlargement (related to Figure 1). (A) As in skin, loss of ADF and CFL1 promotes liver hyperplasia. AhCreADF$^{-/-}$ CFL1$^{fl/fl}$ mice were treated with a single injection of β-naphthoflavone (80 mg/kg) to induce CFL1 gene deletion and aged for 8-9 days post induction. ADF$^{-/-}$ CFL1$^{-/-}$ livers show increased levels of proliferation as evidenced by greater BrdU incorporation or Ki67 staining by immunohistochemistry (IHC). Scale bar 100µm. **(B)** Quantification of BrdU positive nuclei is shown. At least 3 fields per liver were scored. **(C)** Liver weights were measured (g) on dissection and are presented as percent total body weight. The ADF$^{-/-}$ CFL1$^{-/-}$ livers show significant hepatomegaly compared to controls, confirming the enlarged, hardened appearance of the liver on dissection.

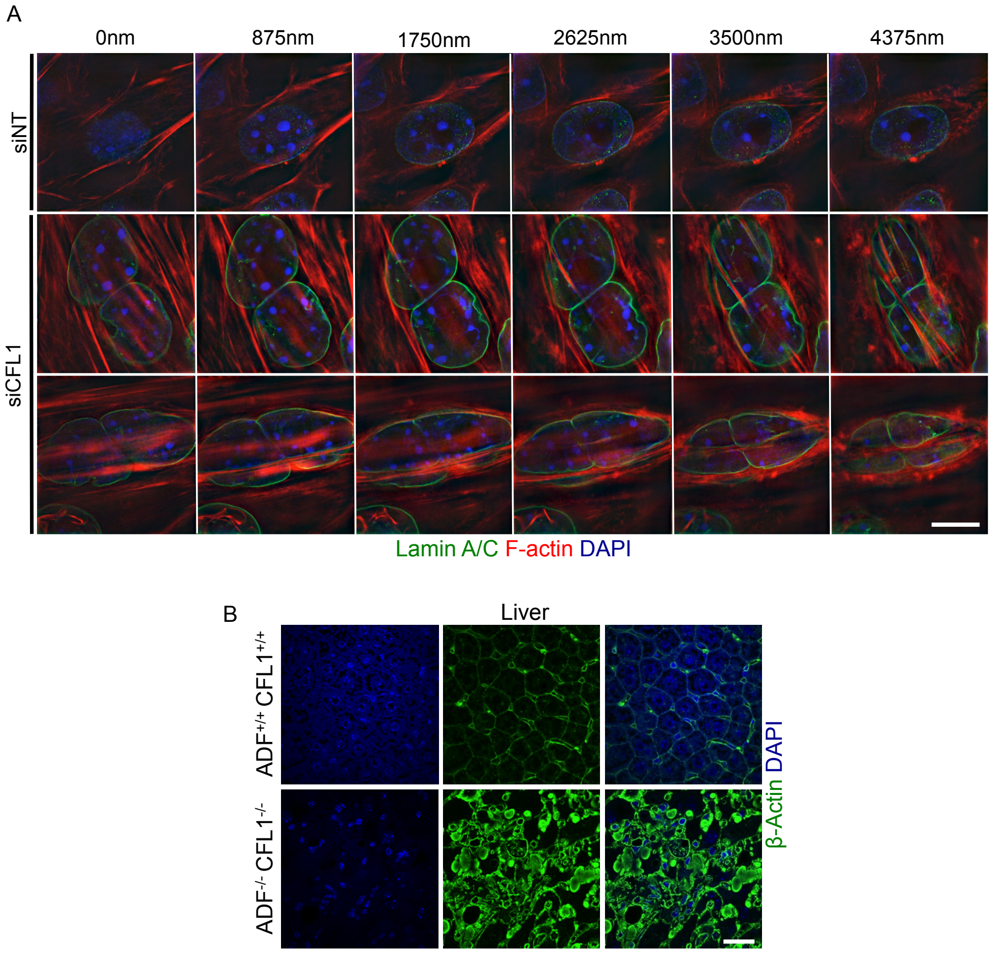

Figure S3. Structured Illumination Microscopy (SIM) and β-actin staining in liver (related to Figure 4). (A) High resolution z-stack of cells treated for 48h with siRNAs for CFL1 or non-targeting, obtained by SIM imaging. Cells stained for Lamin A/C (green), F-actin (red) and DAPI (blue). Scale bar 10μm. **(B)** AhCreADF$^{-/-}$ CFL1$^{fl/fl}$ mice were treated with a single injection of β-naphthoflavone (80 mg/kg) to induce CFL1 gene deletion and aged for 8-9 days post induction. ADF$^{-/-}$ CFL1$^{-/-}$ livers show increased F-actin when stained for β-actin via immunofluorescence (IF). Scale bar 20μm.

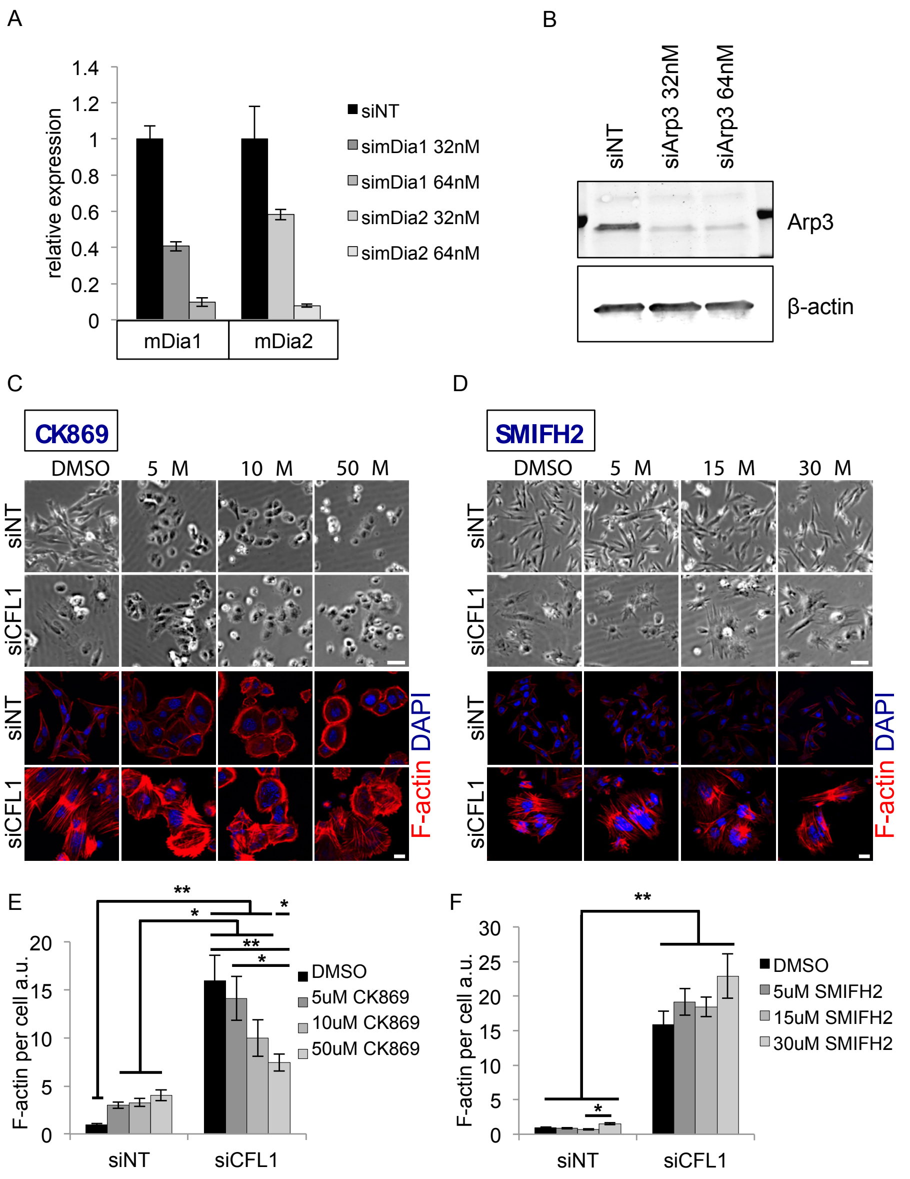

Figure S4. An inhibitor of Arp3, but not formins, suppresses F-actin accumulation following ADF/CFL1 depletion (related to Figure 5). (A) Quantitative PCR was performed to show knock-down of protein expression in cells treated for 72h with 32nM or 64nM of siRNA pools targeting mDia1 and mDia2. The data was normalized against cells treated with 64nM siNT. Bars represent ± S.D. The primers are shown in Table S1. **(B)** Immunoblot for siRNA-mediated knock-down of Arp3. Cells were treated for 72h with two different concentrations of siRNA pools (32nM and 64nM) targeting Arp3. β-actin used as a loading control. **(C-D)** *Upper panels*: Phase contrast images of ADF-null SCCs treated for 30h with siRNAs for siCFL1 or non-targeting siNT, and subsequently incubated overnight with the addition of DMSO or increasing concentrations of inhibitors for Arp2/3 (CK869) or

formins (SMIFH2). *Lower panels*: Cells were stained for F-actin (red) and DAPI (blue). Scale bars 60μm for phase and 25μm for IF images, respectively. **(E-F)** Quantification of F-actin levels per cell was performed using IF confocal images. Cells were stained with phalloidin and measurements were carried out in Image J. ≥18 cells contained in ≥4 fields were quantified per treatment. One representative experiment of two performed is shown. Data presented as mean ± SEM. * One-way ANOVA, p-value ≤ 0.05, ** p-value ≤ 0.002.

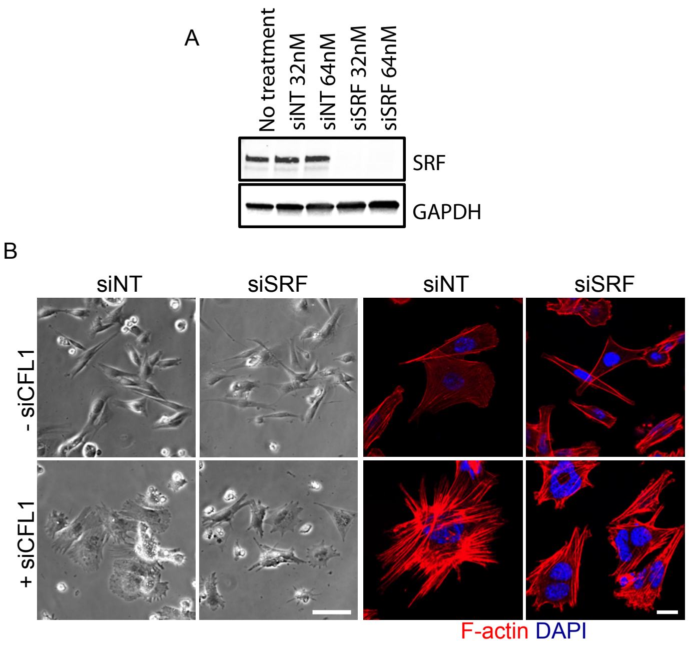

Figure S5. SRF depletion in ADF-null SCC cells (related to Figure 5). (A) Knock-down efficiency of siRNAs targeting SRF was tested by immunoblotting. siRNAs were used in two concentrations, 32nM and 64nM. GAPDH was used as a loading control. **(B)** Phase contrast images of ADF-null SCC cells treated with siNT and siCFL1 alone or alongside siRNAs targeting SRF (siSRF) (left panels). Cells were stained for F-actin (red) and DAPI (blue) (right panels). Scale bars 125μm for phase and 20μm for IF images, respectively.

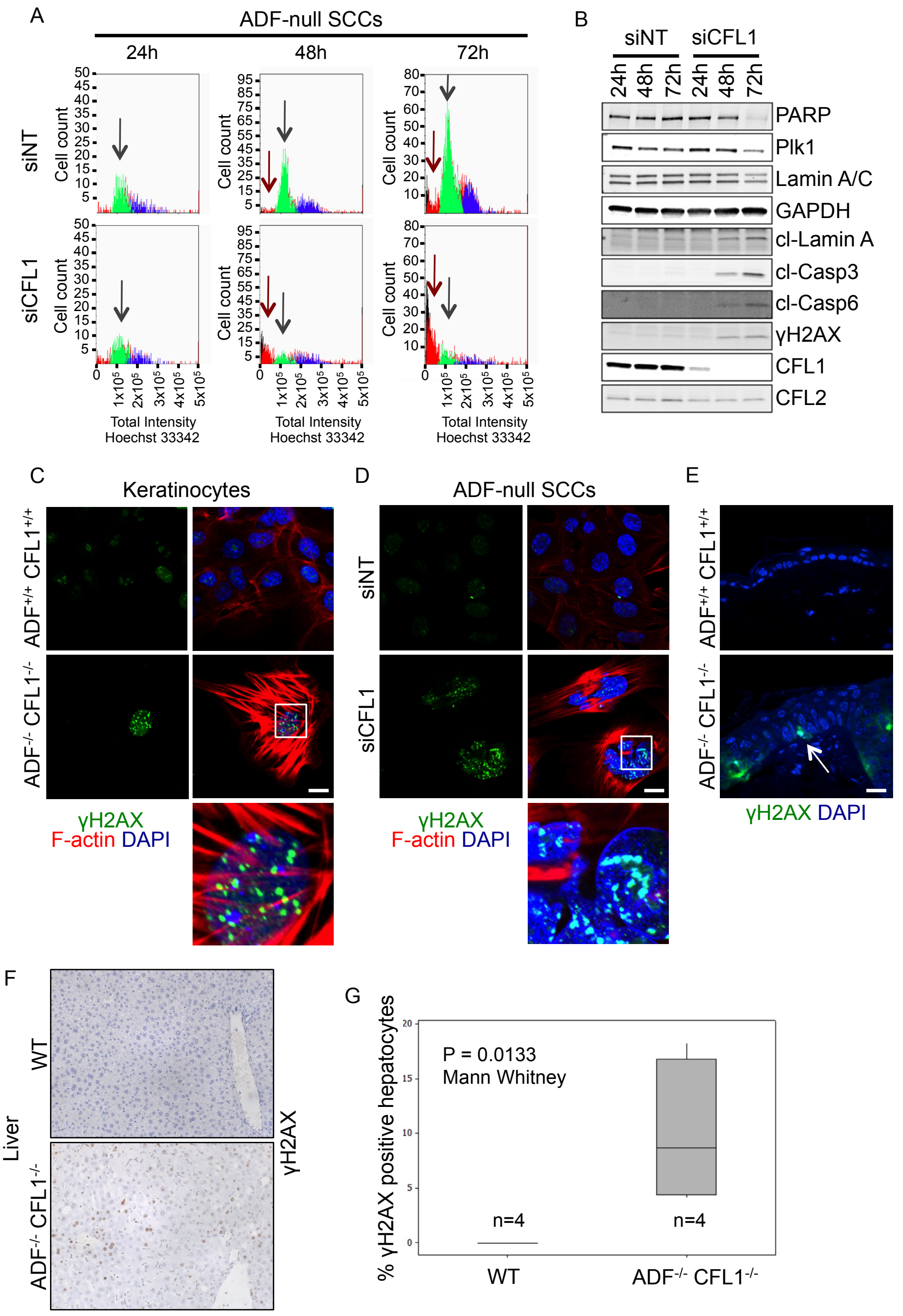

Figure S6. ADF/CFL1 depletion promotes DNA damage and damage-induced signaling (related to Figure 7). (A) Cell cycle profile over 72h of ADF-null SCC cells treated with either siNT or siCFL1. Hoechst 33342 stained nuclei were imaged and analyzed using the Olympus Scan^R acquisition software. Nuclei were gated according to their total intensity, which correlates with total DNA content and subsequently the stage of the cell cycle. Black arrows indicate G1 phase and red arrows show a sub-G1 shift of siRNA treated cells. Experiment repeated twice with

two different cell lines. Pooled data of two cell lines from one representative experiment is shown. **(B)** Immunoblots showing damage-induced apoptotic markers in ADF-null SCC cells treated for 24h, 48h and 72h with siRNAs for CFL1 or non-targeting controls. **(C)** Primary tail keratinocytes isolated from $ADF^{+/+}$ $CFL1^{+/+}$ and $ADF^{-/-}$ $CFL1^{-/-}$ mice were stained with anti-γH2AX (green), TRITC-phalloidin (red) and DAPI (blue). Zoomed image below panel. Scale bar 20μm. **(D)** ADF-null SCCs treated with siNT and siCFL1 for 48h. Cells stained as in (C); zoomed image is provided below panel. Scale bar 20μm. **(E)** Immunofluorescence staining of paraffin-embedded skin sections from $ADF^{+/+}$ $CFL1^{+/+}$ and $ADF^{-/-}$ $CFL1^{-/-}$ mice. Sections stained with anti-γH2AX (green) and DAPI (blue). White arrow indicates DNA-damage associated positive staining in the double knock-out section. Scale bar 20μm. **(F-G)** AhCre$ADF^{-/-}$ $CFL1^{fl/fl}$ mice were treated with a single injection of β-naphthoflavone (80 mg/kg) to induce CFL1 gene deletion and aged for 8-9 days post induction. $ADF^{-/-}$ $CFL1^{-/-}$ livers showed significantly increased levels of γH2AX when stained by IHC. 4 mice were stained for each genotype and at least 3 fields per liver.

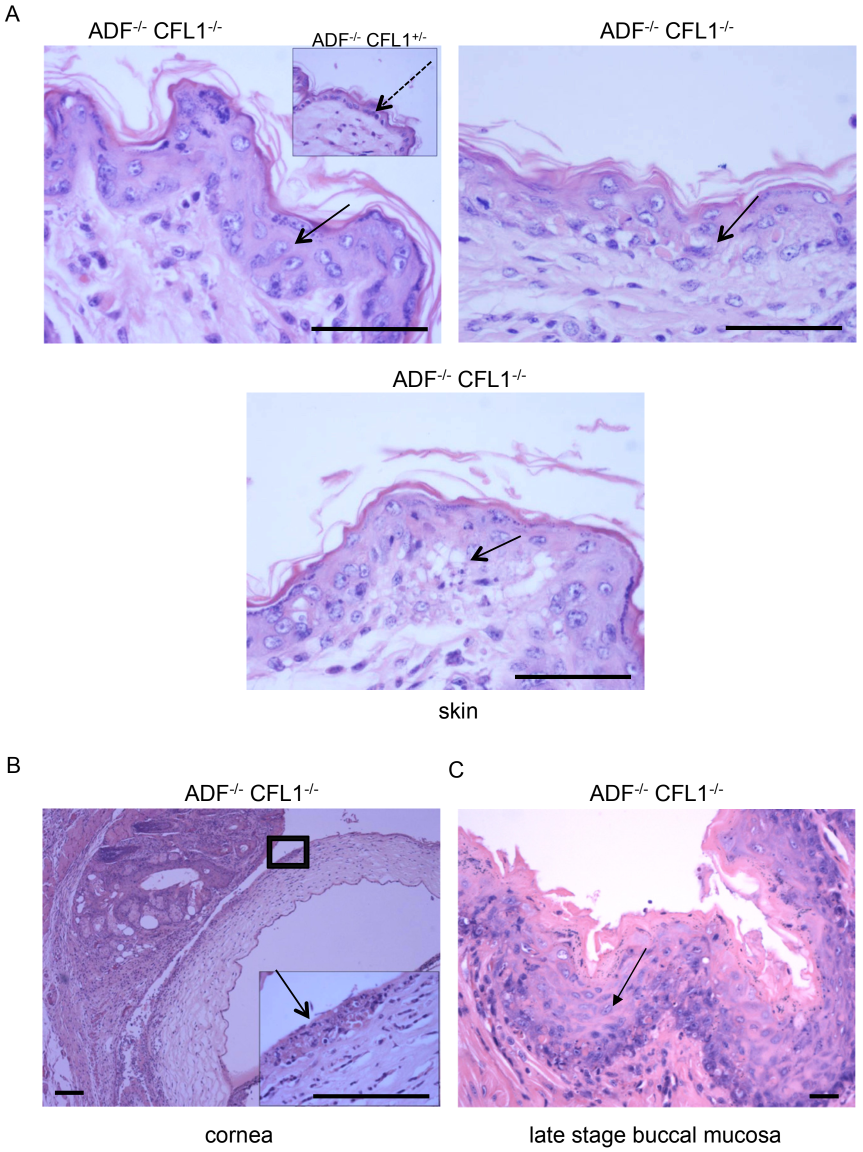

Figure S7. Deletion of ADF and CFL1 causes pathological loss of tissue homeostasis (related to Figure 1 and throughout). (A) Paraffin-embedded skin sections from K14CreER^{T2}/ADF$^{-/-}$/CFL1$^{wt/fl}$ (ADF$^{-/-}$ CFL1$^{+/-}$) (upper left panel, inset) and K14CreER^{T2}/ADF$^{-/-}$/CFL1$^{fl/fl}$ (ADF$^{-/-}$ CFL1$^{-/-}$) mice treated with 4-OHT were stained with hematoxylin and eosin (H & E). Solid arrow in upper left panel indicates presence of hypertrophic epidermis with jumbled keratinocytes while broken arrow shows the comparison with a normal epidermal layer. Solid arrow in upper right panel indicates apoptotic and vacuolated cells within the epidermis while arrow in lower panel shows formation of early vesicles. **(B)** Paraffin-embedded sections of murine cornea from ADF$^{-/-}$ CFL1$^{-/-}$ mice were stained with H & E. Solid arrow in inset zoomed image shows ulcerated corneal epithelium. **(C)** Paraffin-embedded sections of murine buccal mucosa from ADF$^{-/-}$ CFL1$^{-/-}$ mice were stained with H & E. Solid

arrow indicates ulcerative stomatitis. Scale bars 100µm.

Movie S1. ADF/CFL1 depleted cells are capable of membrane protrusion (related to Figure 2E). ADF-null SCCs were treated with non-targeting (siNT) siRNAs. After 48h the cells were imaged every 30 seconds for 30 minutes. This movie provides control for Movie S2.

Movie S2. ADF/CFL1 depleted cells are capable of membrane protrusion (related to Figure 2E). ADF-null SCCs were treated with CFL1 (siCFL1) siRNAs. After 48h the cells were imaged every 30 seconds for 30 minutes. Control transfected with non-targeting (siNT) siRNAs is presented in Movie S1.

Table S1 (Related to Figures 7B, S1E and S4A). List of primers used for quantitative PCR experiments.

target	primer	sequence
Nesprin-2G	forward	tagacgcggacatgcaac
	reverse	ctcaatctctgtcagcatagcc
mDia1	forward	ggtacccgggacaagaaga
	reverse	ctcatgctggtaaatctctcca
mDia2	forward	cctaaaattgctcagaacctttca
	reverse	aatatcatcgtcctgattttctca
ADF	forward	gctttgagaccaaggagtcca
	reverse	tcagaggtgcttgttctggtg
CFL1	forward	tctgtctccctttcgtttcc
	reverse	cattgaacaccttgatgacacc
CFL2	forward	acgtacgaaacaaaagagtctaagaa
	reverse	gcataaatcatcttgctttttaacg
GAPDH	forward	atggtgaaggtcggtgtga
	reverse	aatctccactttgccactgc

SUPPLEMENTAL EXPERIMENTAL PROCEDURES

Generation of transgenic mice

Design, construction, and generation of transgenic mice with either deletion of ADF (Gurniak et al., 2005), targeted loxP sites in the *CFL1* gene (Bellenchi et al., 2007) or expressing the modified Cre recombinase-estrogen receptor fusion under control of the Keratin-14 promoter (K14CreER^{T2}; (Li et al., 2000)) have been described. To enable cell-type-specific CFL1 ablation ADF $^{-/-}$ mice and CFL1$^{fl/fl}$ mice were mated to K14CreER^{T2} transgenic mice. This resulted in offspring that carried K14CreER^{T2} and were nullizygous for ADF and homozygous for the floxed *CFL1* gene (K14CreER^{T2}/ADF$^{-/-}$/CFL1$^{fl/fl}$) or offspring that were nullizygous for ADF but carried two copies of the wild-type *CFL1* allele (K14CreER^{T2}/ADF$^{-/-}$/CFL1$^{wt/wt}$) or offspring that carried two copies of the wild-type *ADF* allele but were homozygous for the floxed *CFL1* gene (K14CreER^{T2}/ADF$^{wt/wt}$/CFL1$^{fl/fl}$). Genotypes were confirmed by PCR analysis. All animals used were crossed onto and maintained on an FVB genetic background. All experiments were carried out in accordance with the UK Coordinating Committee on Cancer Research guidelines by approved protocol (Home Office Project License no. 60/4248).

Preparation and administration of 4-OHT

Tamoxifen (4-OHT) was prepared and administered to 60-day-old K14-ADF mice as described (Indra et al., 1999) except that a reduced dose of 100µg in 100µl of sunflower oil was used. The animals were then left for 10 days before experiments initiated except for K14CreER^{T2}/ ADF$^{-/-}$ CFL1$^{fl/fl}$ mice, which were culled and tissue collected at 4-5 days. 4-OHT for *in vitro* experiments was dissolved in ethanol and used at a final concentration of 10nM for 24-48 hours.

Generation of Squamous Cell Carcinoma cell lines

Ras-driven chemically induced tumours were introduced to mice as previously described (McLean et al., 2001). Squamous Cell Carcinoma (SCC) tumours derived from mice nullizygous to ADF were then removed, cut into small pieces and trypsinised (0.25% in PBS-EDTA, Gibco) for 10min at RT. Trypsin was neutralized with the addition of DMEM + 10% FBS in the presence of 100 Units penicillin and 100µg streptomycin per ml (Sigma). Antibiotics were removed after five passages and

the ADF-null Squamous Cell Carcinoma cells (SCCs) were considered a cell line once reached a stable growing capacity. SCCs were cultured in Dulbecco's Modified Eagle Medium (Sigma) supplemented with 10% FBS (Gibco). All cell cultures were maintained in 37°C, 5% CO_2.

Isolation of primary keratinocytes from mouse tails

Tails were taken from the mice, cut lengthwise and the skin removed. Skins were washed in sterile PBS prior to overnight incubation at 4°C or 2h incubation at 37°C in Dispase (4 mg/ml in PBS, Gibco). The epidermis was removed, cut into several pieces, trypsinized (0.25% in PE, Gibco) for 10min at 37°C and vortexed to dissociate the cells. Cells were re-suspended in Dulbecco's modified Eagle's medium (DMEM, Sigma) + 20% FBS (Gibco) and passed through a 70µm cell strainer (Becton Dickinson). Samples were centrifuged at 1100 rpm for 5 minutes, washed with PBS, centrifuged again and the pellet re-suspended in Keratinocyte Basal Medium (KBM) + KGM singlequotes (Clonetics). Cells were then plated on collagen I-coated cellware (Becton Dickinson) or lysed as described.

CFL1 cloning and overexpression

CFL1 cDNA was cloned into the pWZL-Hygro retroviral vector with a standard DNA ligation kit protocol (Roche). pEco packaging cells were used for viral production and subsequently SCC cells were infected with addition of polybrene (Millipore). Successfully transformed cells were selected using 1:100 Hygromycin (Calbiochem).

Western blotting

Cells were lysed directly in RIPA [50mM Tris–HCl, pH 7.4, 150mM NaCl, 0.1% SDS, 1% NP-40 and 0.5% deoxycholate] with inhibitors (cOmplete ULTRA and phosSTOP tablets, Roche). Clarification was by high-speed centrifugation (13,000 rpm at 4°C for 15min). 20µg of lysates were separated by SDS-PAGE, transferred to nitrocellulose and immunoblotted. Antibodies used were as follows: anti-Destrin (Pierce), anti-Cofilin-1 (CST), anti-Cofilin-2 (m-Cofilin KG-60 from W. Witke or Abcam), anti-Actin/GAPDH/PARP/Myosin-IIA/Lamins/Caspases and γH2AX (all CST), anti-Plk-1 (Millipore) and anti-SUN1 (Santa Cruz). The majority of western blots were analysed using LI-COR Odyssey infrared imaging system and Image Studio software (version 4.0.21). Signal was detected using 680 and 800 IRDye fluorescent antibodies (LI-COR). The software settings were chosen to maximise

signal-to-noise ratio where saturation was not reached and color images generated were converted to grayscale. The blots for β–actin, GAPDH, CFL1 and Plk1 had very low/undetectable background. Western blots presented in Figures 3A and S1A were analysed using traditional luminescence-based detection system.

Immunohistochemistry and BrdU labeling and quantification

Dorsal skin samples were harvested from mice and fixed in neutral-buffered formaldehyde 10% vol/vol (Surgipath Europe), embedded in paraffin and cut into five micron sections for H and E staining or immunohistochemistry. Sections were deparaffinized, rehydrated and unmasked in 10mM citric acid (pH 6.0) in a pressure cooker then stained using DakoCytomation Envision kit appropriate to the species of primary antibody (DakoCytomation Ltd.). The following antibody dilutions were used: anti-Cytokeratin 6 1:1500 and anti-Cleaved Caspase-3 (Asp175) 1:200. All were incubated overnight at 4°C. Sections were counterstained with hematoxylin.

For BrdU labeling mice were injected (ip) with 0.2ml/20g of body weight BrdU (GE Healthcare) 2h prior to harvesting. Skin sections from labeled mice were stained with anti-BrdU antibody (1:1500, CST) as above. Quantification of BrdU positive nuclei from the mouse epidermis was performed with ImmunoRatio, a web-based application for image analysis (Tuominen et al., 2010).

Immunofluorescence

Sections were deparaffinized, rehydrated and unmasked in 10mM citric acid (pH 6.0) in a pressure cooker for 10 minutes. They were then blocked for 1 hour with 1% BSA/2% FBS in water (Blocking buffer). Primary antibodies diluted in Blocking buffer were incubated for 1 hour, washed 3 times for 5min in 0.1% Tween 20/TBS (TBST) and Alexa Fluor-488- or Alexa Fluor-594-conjugated secondary antibodies (Invitrogen) added for 1 hour RT. Sections rinsed 3 times for 5min in TBST and mounted with Vectashield Mounting Media containing DAPI (Vector Laboratories). For β-actin and Lamin A/C staining dorsal skin samples were harvested and fixed for 30 minutes at 37°C in 4% paraformaldehyde, cooled down to RT and incubated overnight at 4°C before being embedded in paraffin. 5μm sections were deparaffinized, rehydrated and unmasked as described above before being blocked with peroxidase and protein blocking buffers for 2h at RT (DakoCytomation) and incubated overnight at 4°C with primary antibodies.

The following antibody dilutions were used: anti-E-cadherin 1:100 (CST), anti-Cytokeratin 14 1:100 (made in-house), anti-Cytokeratin 10 1:1500, anti-Cytokeratin 6 1:1500 (Abcam), anti-β-actin and Lamin A/C 1:200 (CST).

Keratinocytes and SCCs were grown on glass coverslips, fixed and permeabilized at room temperature for 10 min (3.7% formaldehyde, 0.2% Triton X-100, 1mM $MgCl_2$, 100mM PIPES and 10mM EGTA), blocked for 1 hour (2% BSA in 0.1% Triton X-100/TBS) and incubated with primary antibodies overnight at 4°C. Antibody detection was via Alexa Fluor-488 or Alexa Fluor-594-conjugated secondary antibodies for 45 min at RT. Between steps, cells were washed with 0.1% Triton X-100/TBS.

The following antibody dilutions were used: anti-E-cadherin 1:100, Lamin A/C and Myosin-IIA 1:50, pMLC2 (Ser19) 1:200, p-paxillin 1:50, α-actinin 1:50, γH2AX 1:400 (all CST) and anti-Vinculin 1:100 (Abcam). F-actin was visualized using TRITC-Phalloidin (Sigma) or Rhodamine Phalloidin (Life Technologies) along with the secondary antibody mix.

All immunofluorescent experiments were visualized using an Olympus FV1000 confocal microscope equipped with an UPLANSAPO 60X/1.35 oil immersion objective. Super-resolution imaging was performed with Nikon's N-SIM super-resolution microscope equipped with 100x oil objective lens (N.A. 1.49).

RNA interference and protein co-depletions

siRNA reverse transfections were carried out using RNAiMAX lipofectamine reagent (Life Technologies) according to the company's standard protocol. Non-targeting and CFL1 targeting siRNAs (Dharmacon) were used at a final concentration 32nM. CFL1 knock-down experiments carried out for 48h. siRNAs for Arp3, mDia1, mDia2, SRF and Nesprin-2G (Dharmacon) were used at final concentration 64nM.

For all co-depletion experiments cells were treated with siRNAs targeting the proteins to be depleted alongside CFL1 at day 1. 24h later media was changed and cells were treated for further 48h with a mixture of siRNAs targeting CFL1 and the co-depleted protein. Control cells were treated either with non-targeting siRNAs alone or alongside siRNAs for the co-depleted proteins.

Live cell imaging, kymograph and membrane dynamics analysis

48h post siRNA treatment cells were imaged every 30 seconds for 30 minutes using a Scan^R high content screening station with a 20X phase objective (Olympus). Kymograph and membrane analysis were performed as previously described (Benjamin et al., 2010). Kymograph profiles were compiled using normalized pixel intensities from 1-pixel wide kymographs. Protrusion length was calculated based on the kymograph peaks and protrusion persistence was calculated from the peaks' width, representing the time for a full membrane extension and retraction.

Inhibitor treatments

Prior to treatment with inhibitors cells were transfected with siRNAs for CFL1 or non-targeting. 30h post transfection media was changed with fresh containing the inhibitors and cells were incubated overnight prior to fixation.

Free barbed–ends assay and ImageJ quantification

Free barbed-end formation was assessed and quantified by an adaptation of the method described previously (Worth et al., 2010). Cells plated on coverslips were permeabilized using 0.125 mg/ml saponin in the presence of 9μg biotin conjugated G-actin (Cytoskeleton). They were incubated for 2min in 37°C and subsequently fixed with the standard formaldehyde fixation protocol described above. Detection of biotinylated actin was performed using FITC-streptavidin (20μg/ml, Vector) and F-actin was labelled with TRITC-phalloidin (Sigma). Images were acquired with an Olympus FV-1000 confocal microscope equipped with an UPLANSAPO 60X/1.35 oil immersion objective. The laser and acquisition settings were kept the same for imaging every condition, ensuring that there was no pixel saturation. Quantification of total pixel intensity per cell was performed using ImageJ after background subtraction for each field individually. Same method was used for the quantification of F-actin.

G- and F-actin assay

For G-actin isolation 60mm dishes of cells treated with siRNAs were rinsed twice with CSK buffer (10mM PIPES pH 6.8, 50mM NaCl, 3mM $MgCl_2$, 300mM Sucrose). Then, 500μl of lysis buffer 1 were added (CSK buffer + 1% Triton X-100, 1μg/ml phalloidin, protein inhibitors (cOmplete and phosSTOP tablets (Roche))), and plates incubated for 5min at RT on shaker. Supernatants were then collected and SDS was added to a final concentration of 2%.

F-actin was isolated after washing the dishes three times with CSK buffer and

incubating 5min at RT on shaker with 500µl of lysis buffer 2 (Lysis Buffer 1 + 2% SDS). The cells were then scraped and the samples were homogenized with syringes and 25 gauge needles. Protein assay was done on G-actin sample and equivalent amounts of lysates were run on a 4-12% polyacrylamide gel.

Cell cycle analysis

Cell cycle analysis was image based using the Scan^R high content screening station (Olympus). Cells were classified in G1 or S/G2/M phases according to their nuclear intensity which depends on the nuclear content. Nuclei were stained for 1h at 37°C with Hoechst 33342 (Invitrogen) prior to imaging. Image analysis was performed with the ScanR Image Analysis software.

Deletion of genes encoding ADF and CFL1 in mouse liver

AhCre^{+} mice (Ireland et al., 2004) were intercrossed with ADF$^{-/-}$/CFL1$^{fl/fl}$ mice. To induce recombination mice were given a single injection of 80mg/kg ß-naphthoflavone (IP). For BrdU labeling, mice were injected 250µl BrdU (GE Healthcare) 2h prior to harvesting. Staining was performed using an anti-BrdU antibody (BD Bioscience) at 1:200 or Ki-67 (Thermo) at 1:100 on formalin fixed liver sections.

Quantitative RT-PCR

RNA was extracted from cells using the RNeasy kit (QIAGEN) and then converted to cDNA with SuperScript First-Strand cDNA synthesis kit with Random Hexamer primers (Invitrogen). qPCR reactions were prepared with the SensiFAST Hi-ROX kit (Bioline) and run on StepOnePlus Real Time PCR system (Life Technologies). All procedures were done according to manufactures standard protocols. Results were analyzed with ΔΔCt method using GAPDH as endogenous control, and statistical significance was assessed with Student's t-test. All primers were designed in Universal ProbeLibrary Assay Design Center (Roche) and can be found in **Table S1**.

Statistical analysis

Statistical analysis was performed in Prism or in Excel for unpaired t-tests. Normality of the data of every experiment was assessed with D'Agostino & Pearson's normality test. When data was fitted in a normal distribution, statistical significance was assessed by Student's t-test in case of two groups, or by One-way ANOVA and

Tukey's multiple comparisons test for bigger groups. If data was not normally distributed, then Mann-Whitney's or Kruskal–Wallis one-way analysis of variance accompanied by Dunn's multiple comparisons test were utilised to ascertain statistical significance between two or more groups accordingly. Statistical significance was assumed for p-values < 0.05. Sample sizes, replicates and test used are indicated for each experiment in the figure legends.

SUPPLEMENTAL REFERENCES

Ireland, H., Kemp, R., Houghton, C., Howard, L., Clarke, A.R., Sansom, O.J., and Winton, D.J. (2004). Inducible Cre-mediated control of gene expression in the murine gastrointestinal tract: effect of loss of beta-catenin. Gastroenterology *126*, 1236-1246.

McLean, G.W., Brown, K., Arbuckle, M.I., Wyke, A.W., Pikkarainen, T., Ruoslahti, E., and Frame, M.C. (2001). Decreased focal adhesion kinase suppresses papilloma formation during experimental mouse skin carcinogenesis. Cancer Res. *61*, 8385-8389.

Tuominen, V.J., Ruotoistenmaki, S., Viitanen, A., Jumppanen, M., and Isola, J. (2010). ImmunoRatio: a publicly available web application for quantitative image analysis of estrogen receptor (ER), progesterone receptor (PR), and Ki-67. Breast Cancer Res. *12*.

Worth, D.C., Hodivala-Dilke, K., Robinson, S.D., King, S.J., Morton, P.E., Gertler, F.B., Humphries, M.J., and Parsons, M. (2010). alpha nu beta 3 integrin spatially regulates VASP and RIAM to control adhesion dynamics and migration. J. Cell Biol. *189*, 369-383.
