# Supplementary figures and images for: ADF and Cofilin1 Control Actin Stress Fibers, Nuclear Integrity, and Cell Survival
Source: Cell Rep. 2015 Nov 19;13(9):1949–64. doi: 10.1016/j.celrep.2015.10.056 (PMC4678118; doi:10.1016/j.celrep.2015.10.056)

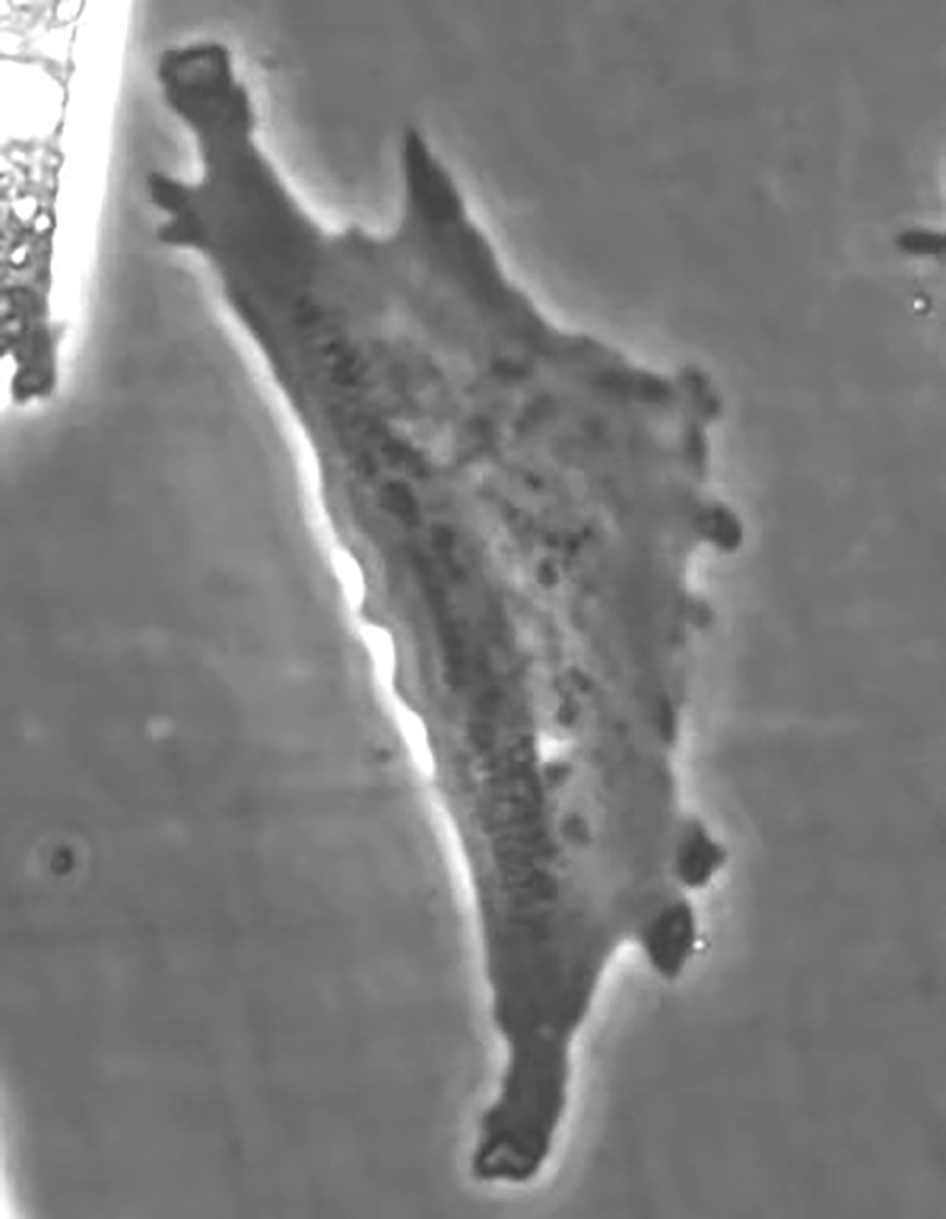

Supplement: Movie S1. ADF/CFL1-Depleted Cells Are Capable of Membrane Protrusion, Related to Figure 2E — ADF-null SCCs were treated with non-targeting (siNT) siRNAs. After 48h the cells were imaged every 30 seconds for 30 minutes. This movie provides control for Movie S2. [file mmc2.jpg]

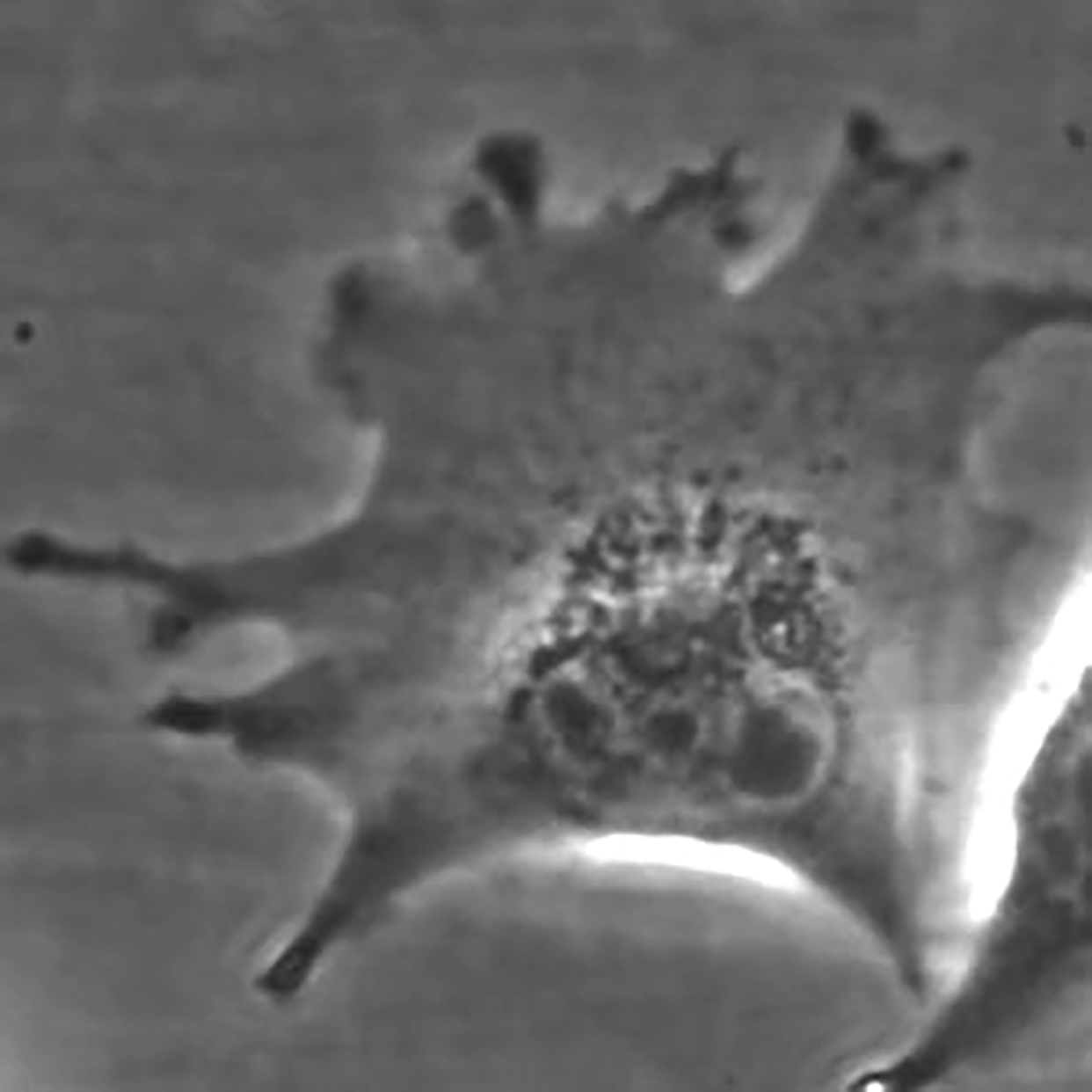

Supplement: Movie S2. ADF/CFL1-Depleted Cells Are Capable of Membrane Protrusion, Related to Figure 2E — ADF-null SCCs were treated with CFL1 (siCFL1) siRNAs. After 48h the cells were imaged every 30 seconds for 30 minutes. Control transfected with non-targeting (siNT) siRNAs is presented in Movie S1. [file mmc3.jpg]
