# Supplementary material for: ADF and Cofilin1 Control Actin Stress Fibers, Nuclear Integrity, and Cell Survival
Source: Cell Rep. 2015 Nov 19;13(9):1949–64. doi: 10.1016/j.celrep.2015.10.056 (PMC4678118; doi:10.1016/j.celrep.2015.10.056)
Supplement: Document S2. Article plus Supplemental Information [file mmc4.pdf]

Cell Reports

Article

ADF and Cofilin1 Control Actin Stress Fibers, Nuclear Integrity, and Cell Survival

Graphical Abstract

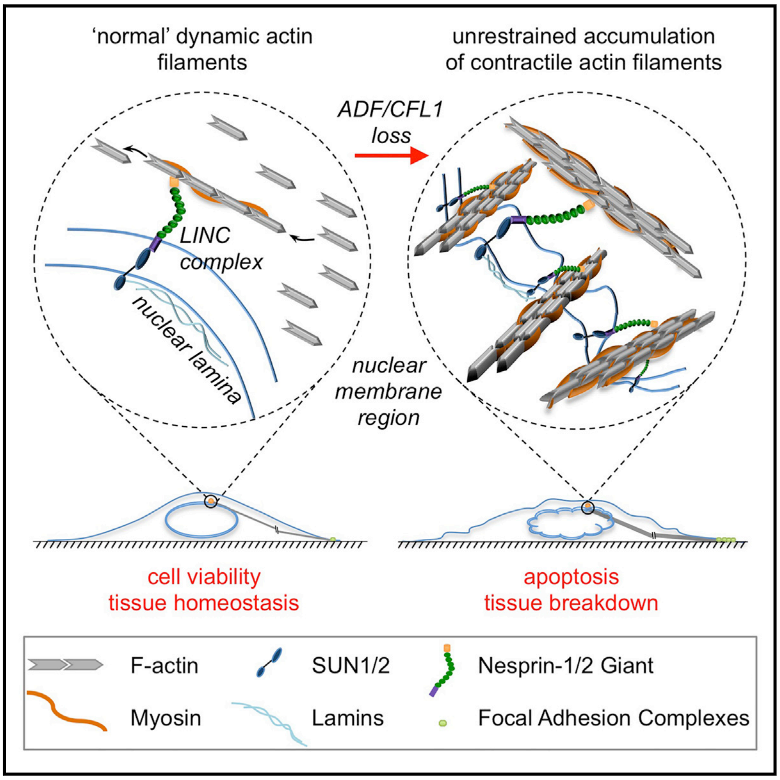

Highlights

- Loss of both ADF and CFL1 severely perturbs adult tissue homeostasis
- ADF/CFL1 loss results in uncontrolled accumulation of contractile actin stress fibers
- Acto-myosin tension causes nuclear deformation mediated by the LINC complex
- ADF and CFL1 control nuclear integrity and cell survival via actin dynamics

Authors

Georgios Kanellos, Jing Zhou, Hitesh Patel, ..., Walter Witke, Valerie G. Brunton, Margaret C. Frame

Correspondence

m.frame@ed.ac.uk

In Brief

The filament-severing proteins ADF and CFL1 mediate actin stress fiber turnover. Kanellos et al. find that their co-depletion leads to uncontrolled accumulation of contractile actin filaments, which, via the LINC complex, physically disrupt the nucleus and cause loss of cell and tissue viability.

Kanellos et al., 2015, Cell Reports *13*, 1949–1964
December 1, 2015 ©2015 The Authors
http://dx.doi.org/10.1016/j.celrep.2015.10.056

Cell Reports
Article

OPEN ACCESS
CellPress

ADF and Cofilin1 Control Actin Stress Fibers, Nuclear Integrity, and Cell Survival

Georgios Kanellos,[1,4] Jing Zhou,[1,4,5] Hitesh Patel,[1,4] Rachel A. Ridgway,[2] David Huels,[2] Christine B. Gurniak,[3] Emma Sandilands,[1] Neil O. Carragher,[1] Owen J. Sansom,[2] Walter Witke,[3] Valerie G. Brunton,[1] and Margaret C. Frame[1,*]
[1]Edinburgh Cancer Research UK Centre, Institute of Genetics and Molecular Medicine, University of Edinburgh, Western General Hospital, Crewe Road South, Edinburgh EH4 2XR, UK
[2]Cancer Research UK Beatson Institute, Garscube Estate, Switchback Road, Bearsden, Glasgow G61 1BD, UK
[3]Institute of Genetics, University of Bonn, Karlrobert-Kreiten Strasse 13, 53115 Bonn, Germany
[4]Co-first author
[5]Present address: Department of General Surgery, Union Hospital, Tongji Medical College, Huazhong University of Science and Technology, 1277 Jiefang Avenue, Wuhan, Hubei 430022, China
*Correspondence: m.frame@ed.ac.uk
http://dx.doi.org/10.1016/j.celrep.2015.10.056
This is an open access article under the CC BY license (http://creativecommons.org/licenses/by/4.0/).

SUMMARY

Genetic co-depletion of the actin-severing proteins ADF and CFL1 triggers catastrophic loss of adult homeostasis in multiple tissues. There is impaired cell-cell adhesion in skin keratinocytes with dysregulation of E-cadherin, hyperproliferation of differentiated cells, and ultimately apoptosis. Mechanistically, the primary consequence of depleting both ADF and CFL1 is uncontrolled accumulation of contractile actin stress fibers associated with enlarged focal adhesions at the plasma membrane, as well as reduced rates of membrane protrusions. This generates increased intracellular acto-myosin tension that promotes nuclear deformation and physical disruption of the nuclear lamina via the LINC complex that normally connects regulated actin filaments to the nuclear envelope. We therefore describe a pathway involving the actin-severing proteins ADF and CFL1 in regulating the dynamic turnover of contractile actin stress fibers, and this is vital to prevent the nucleus from being damaged by actin contractility, in turn preserving cell survival and tissue homeostasis.

INTRODUCTION

The cofilin family of actin depolymerizing factor proteins controls actin dynamics by severing and depolymerizing actin filaments (reviewed in Mizuno, 2013). There are three highly conserved cofilins (between 70% and 81% identical at the amino acid level); these are Cofilin-1 (CFL1; also known as non-muscle- or n-Cofilin), ADF (stands for actin-depolymerizing factor; also known as Destrin), and Cofilin-2 (CFL2; also known as muscle- or m-Cofilin). These have distinct but overlapping expression patterns and are considered to have similar biochemical functions; they bind actin monomers and filaments (G-actin and F-actin, respectively; Lappalainen and Drubin, 1997). Their activities increase the number of actin monomers and filament fragments, so permitting filament turnover and treadmilling at key locations in migrating cells (reviewed in Bugyi and Carlier, 2010).

Despite a huge literature on the role of cofilin(s) in actin treadmilling and cell migration in vitro and in the behavior of cancer cells associated with invasion (DesMarais et al., 2005; Wang et al., 2007), genetic co-deletion of both actin-severing cofilins in adult tissues has not been carried out to address what are their fundamental roles in overall cellular actin regulation and the consequences for cell and tissue homeostasis. Data to date imply that ADF and CFL1 likely have some distinct and some overlapping functions in vivo. CFL1-deficient mice are not viable, dying at E11.5–12.5 due to aberrant neural tube closure and defective neural crest cell migration (Gurniak et al., 2005). ADF is therefore unable to compensate for loss of CFL1 during embryonic development, although it is highly expressed in the cranial neuroectoderm (Gurniak et al., 2005). ADF-deficient mice are viable, with normal brain appearance, but suffer from corneal defects in adult mice that cause blindness (Bellenchi et al., 2007; Ikeda et al., 2003). Conditional loss of CFL1 in neuronal cells causes over-differentiation, altered proliferation, and migration that are linked to a lissencephaly phenotype (Bellenchi et al., 2007). In ureteric bud, loss of function of both CFL1 and ADF arrests branching morphogenesis, implying functional redundancy in this context (Kuure et al., 2010).

Here, we address the fundamental roles of ADF and CFL1, the most-potent actin-severing cofilins (Vartiainen et al., 2002), in adult cells and tissues, demonstrating dynamic stress fiber regulation required for maintenance of nuclear shape and integrity, cell survival, and adult tissue homeostasis. Depletion of ADF and CFL1 triggered accumulation of aberrant, contractile actin fibers that increased intracellular tension, leading to actin-dependent nuclear deformation via the LINC complex that connects the actin cytoskeleton to the nuclear lamina. Thus, redundant roles of ADF and CFL1 include to dynamically control tensile actin stress fibers and focal adhesions, and this is vital for maintenance of nuclear shape, nuclear integrity, and cell and tissue viability.

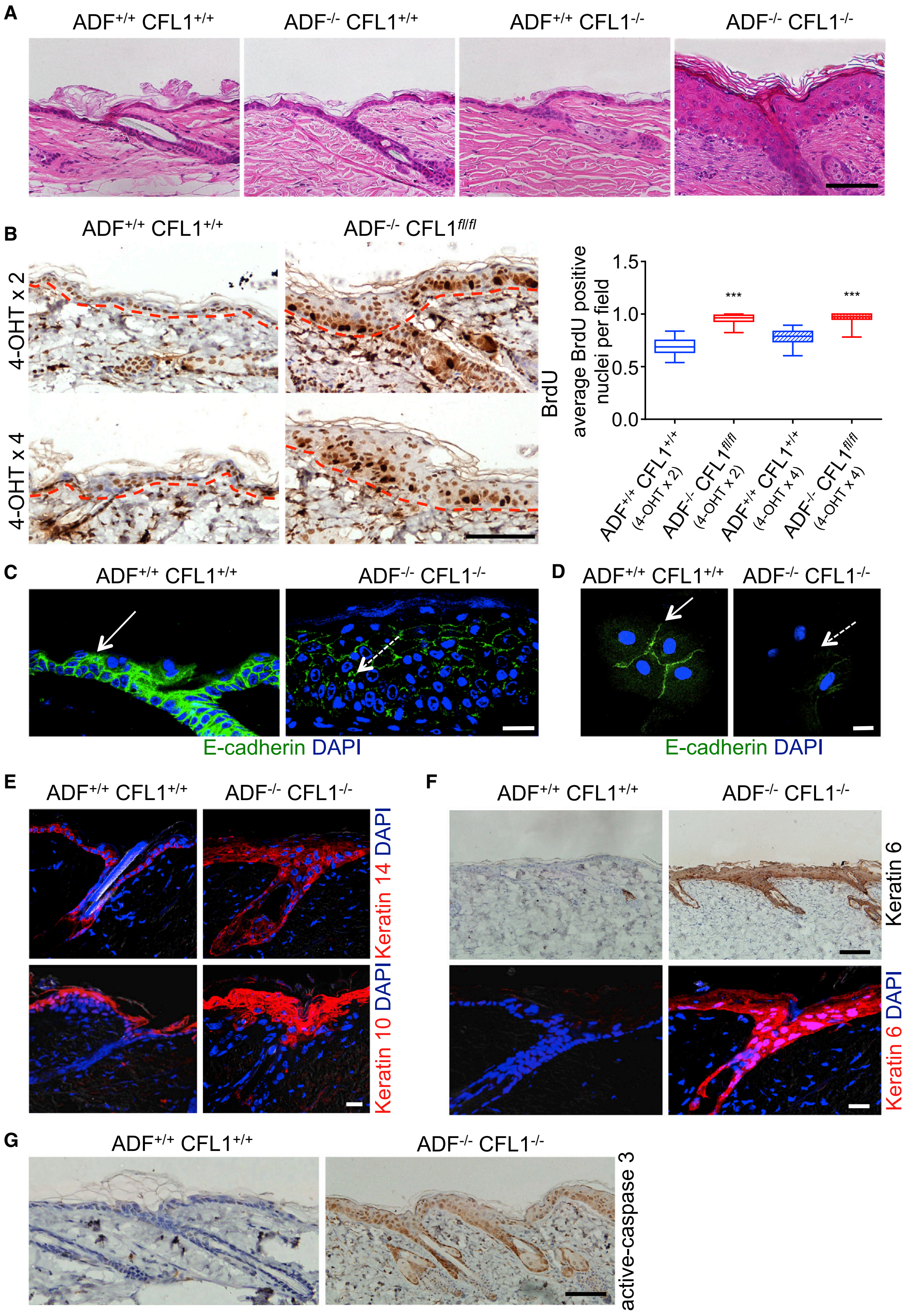

(legend on next page)

RESULTS

Knockout of ADF and CFL1 Promotes Loss of Tissue Homeostasis

In order to study the role of ADF and CFL1, the two main actin-severing forms of cofilin in epithelial cells (Vartiainen et al., 2002), we intercrossed K14CreER^{T2} mice with ADF$^{-/-}$ mice and with mice expressing CFL1 flanked with loxP sites (CFL1$^{fl/fl}$) or with mice that were both ADF$^{-/-}$ and CFL1$^{fl/fl}$ (Figures 1 and S1A). K14CreER^{T2}, K14CreER^{T2}/ADF$^{-/-}$/CFL1$^{WT/WT}$, K14CreER^{T2}/ADF$^{WT/WT}$/CFL1$^{fl/fl}$, and K14CreER^{T2}/ADF$^{-/-}$/CFL1$^{fl/fl}$ mice were treated with tamoxifen (4-OHT), as we have described previously (McLean et al., 2001). This permitted us to examine the effects of deleting one or both ADF and CFL1 isoforms from cells in the epidermis of adult mice.

We found that skin from K14CreER^{T2} mice expressed both ADF and CFL1 (referred to hereafter as ADF$^{+/+}$ CFL1$^{+/+}$), whereas skin from K14CreER^{T2}/ADF$^{-/-}$/CFL1$^{WT/WT}$ mice was completely deficient in ADF (referred to as ADF$^{-/-}$ CFL1$^{+/+}$; Figure S1A). 4-OHT-treated skin from K14CreER^{T2}/ADF$^{WT/WT}$/CFL1$^{fl/fl}$ mice expressed ADF but greatly reduced CFL1 (referred to as ADF$^{+/+}$ CFL1$^{-/-}$), whereas 4-OHT-K14CreER^{T2}/ADF$^{-/-}$/CFL1$^{fl/fl}$ mice expressed no detectable ADF and little CFL1 (referred to as ADF$^{-/-}$ CFL1$^{-/-}$; Figures 1 and S1A). Interestingly, CFL2 was present in all mice irrespective of genotype, but its expression in the skin was apparently unaltered by the loss of ADF and/or CFL1 (Figure S1A).

Upon staining skin sections with H&E, we observed that ADF$^{-/-}$ CFL1$^{+/+}$ or ADF$^{+/+}$ CFL1$^{-/-}$ skin appeared normal and similar to wild-type (WT) ADF$^{+/+}$ CFL1$^{+/+}$ skin (Figure 1A). However, when both ADF and CFL1 isoforms were depleted in ADF$^{-/-}$ CFL1$^{-/-}$ skin, there was a striking thickening of the epidermis and an apparent accumulation of cells at day 5 following treatment with 4-OHT (Figure 1A). Expression of CFL2 was apparently unable to compensate for loss of ADF and CFL1, indicating that only the redundant functions of ADF and CFL1 regulate normal skin homeostasis. We presume this is a function of actin severing, because CFL2 has much-weaker actin-severing activity (Vartiainen et al., 2002).

To characterize the reasons for thickening of the epidermis, we first determined whether depletion of ADF/CFL1 affected cell proliferation. K14CreER^{T2} and K14CreER^{T2}/ADF$^{-/-}$/CFL1$^{fl/fl}$ mice were treated for 2 or 4 days with 4-OHT and labeled with BrdU 2 hr prior to harvesting of skin. In the absence of ADF and CFL1, we observed a significant increase in the number of keratinocytes actively replicating their genomes compared to their WT counterparts. The number of proliferating epidermal cells increased to a maximum irrespective of whether 4-OHT was administered for 2 or 4 days, implying that the consequences of Cre-induced *CFL1* excision from the mouse epidermis was a rapid effect (Figure 1B).

To establish whether this phenotype was recapitulated in other tissues, we also deleted ADF and CFL1 within the hepatocytes of the adult liver using an Ah-Cre transgene. Following a single injection of the cytochrome p450 inducer β-napthoflavone, Cre is induced in approximately 90% of hepatocytes (Reed et al., 2008). By 9 days after gene deletion, these mice developed very obvious hepatomegaly that was associated with increased BrdU and Ki67 staining (Figures S2A–S2C), and the mice had to be sacrificed.

We next addressed whether there was a difference in the morphology of the epithelial cells present in the ADF/CFL1 knockout mice. Using immunofluorescence (IF), we observed that ADF$^{+/+}$ CFL1$^{+/+}$ skin cells had a typical epithelial morphology with E-cadherin clearly visible in cell-cell contacts (Figure 1C). However, E-cadherin was barely visible at cell-cell adhesions of doubly depleted ADF$^{-/-}$ CFL1$^{-/-}$ skin (Figure 1C). In the presence of ADF and CFL1, primary cultures of keratinocytes also have visible cell-cell contacts, whereas keratinocytes isolated from double ADF/CFL1 knockout skin display reduced membrane E-cadherin staining (Figure 1D) and exhibit a more-mesenchymal-like, elongated, and flattened morphology (see later in Figure 2A). These data support a critical role of ADF/CFL1 in cell morphology and epithelial tissue organization.

To further examine the expanded cell population after ADF/CFL1 loss, we stained skin sections with markers of differentiation. Both keratin (K)14 and K10 were expressed throughout the thickened epidermis (Figure 1E), indicating that hyperproliferation and differentiation occurred concomitantly in the absence of ADF and CFL1. K6 is a marker of “active keratinocytes” that are proliferating, migratory cells identified in wound healing and other pathological conditions (Freedberg et al., 2001). We found that K6 expression was increased throughout the thickened epidermis upon ADF/CFL1 co-depletion (Figure 1F), implying that there was aberrant induction of the keratinocyte activation cycle normally associated with inflammatory and/or wound repair signaling. Loss of ADF and CFL1 also resulted in increased expression of the cleaved form of caspase-3 in vivo, indicating a high spontaneous level of apoptosis (Figure 1G). Taken together, these data demonstrate that overlapping

Figure 1. Deletion of ADF and CFL1 Causes Epidermal Thickening and Loss of Tissue Homeostasis

(A) Paraffin-embedded (PE) skin sections from K14CreER^{T2} (ADF$^{+/+}$ CFL1$^{+/+}$), K14CreER^{T2}/ADF$^{-/-}$/CFL1$^{WT/WT}$ (ADF$^{-/-}$ CFL1$^{+/+}$), K14CreER^{T2}/ADF$^{WT/WT}$/CFL1$^{fl/fl}$ (ADF$^{+/+}$ CFL1$^{-/-}$), and K14CreER^{T2}/ADF$^{-/-}$/CFL1$^{fl/fl}$ (ADF$^{-/-}$ CFL1$^{-/-}$) mice treated with 4-OHT were stained with H&E. The scale bar represents 100 μm.

(B) BrdU staining of PE skin sections from K14CreER^{T2} (ADF$^{+/+}$ CFL1$^{+/+}$)- and K14CreER^{T2}/ ADF$^{-/-}$/CFL1$^{fl/fl}$ (ADF$^{-/-}$ CFL1$^{fl/fl}$)-labeled mice, treated for 2 or 4 days with 4-OHT (4-OHT × 2 and 4-OHT × 4, respectively). The scale bar represents 100 μm. Quantification is shown (right panel), with box plots displaying the full range of variation (min to max). Greater than or equal to seven fields/mouse were quantified for two mice/treatment/genotype. ***Mann-Whitney p value < 0.0001.

(C and D) Immunofluorescent (IF) staining for E-cadherin of skin sections and keratinocytes isolated from mouse tails. Solid arrows indicate the presence of E-cadherin in cell-cell contacts whereas broken arrows indicate its absence. The scale bars represent 20 μm.

(E) IF staining of skin sections for Keratin 14 or Keratin 10. The scale bar represents 20 μm.

(F) Immunohistochemical (IHC) (upper panels) and IF staining (lower panels) for Keratin 6. The scale bars represent 100 μm for IHC and 20 μm for IF.

(G) IHC of PE skin sections for cleaved caspase 3. The scale bar represents 100 μm.

Nuclei were counterstained with DAPI in IF images. See also Figures S1A, S2, and S7.

OPEN ACCESS
CellPress

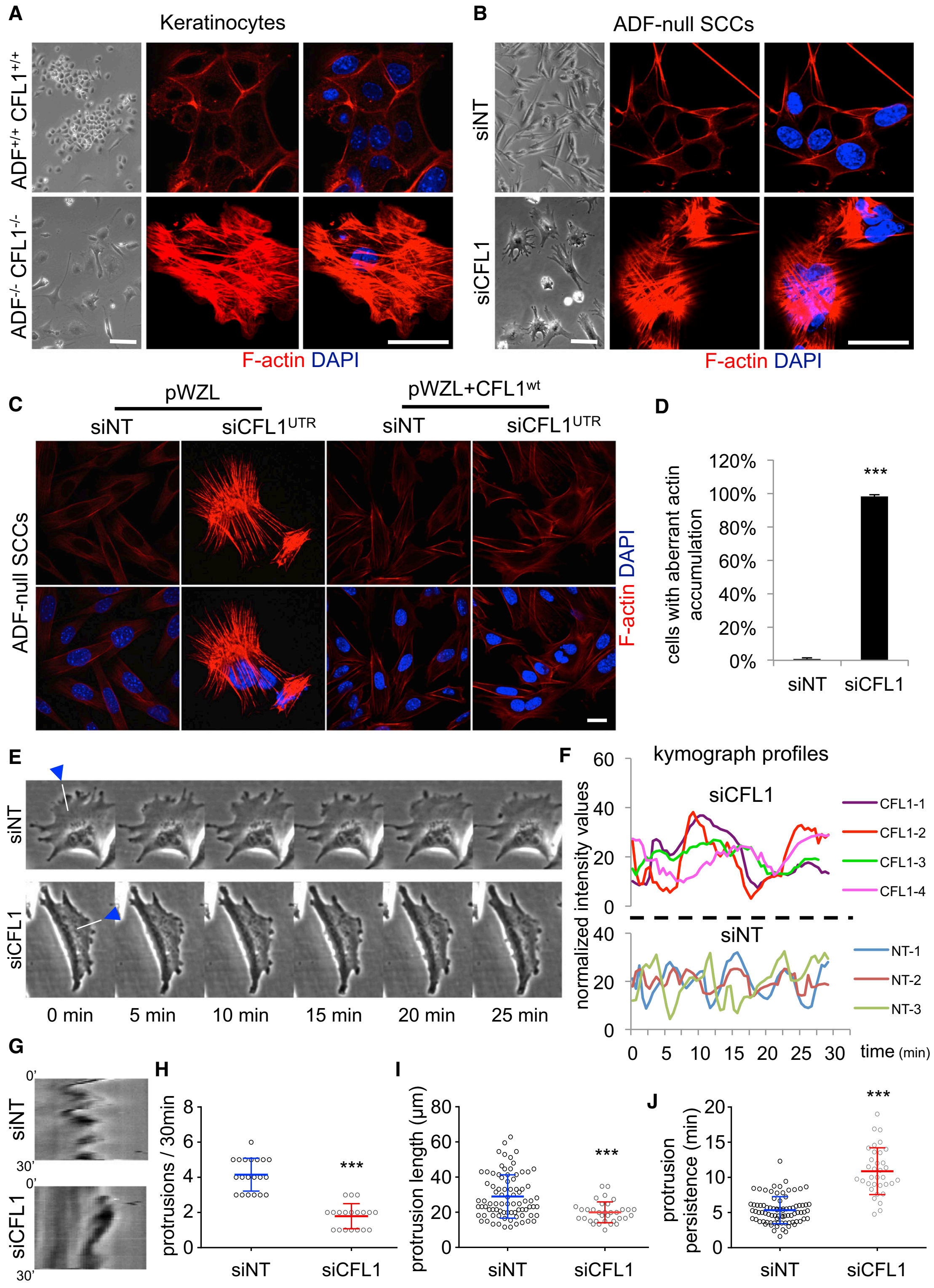

Figure 2. ADF/CFL1 Loss Causes Stress Fiber Accumulation

(A) Phase contrast images of keratinocytes (left panels) and fluorescent staining for filamentous actin (F-actin) (right panels).

(B) Squamous cell carcinoma cells (SCCs) were obtained from ADF$^{-/-}$ CFL1$^{+/+}$ mice (ADF-null SCCs). Due to lethality, double knockout SCCs could not be propagated. The remaining isoform (CFL1) was transiently knocked down with siRNAs (siCFL1). Phase contrast images of ADF-null cells treated with non-targeting siRNAs (siNT) or siRNAs for CFL1 (siCFL1) are shown (left panels). F-actin is visualized by fluorescence in right panels.

(legend continued on next page)

ADF/CFL1 activity is absolutely required for the maintenance of normal tissue homeostasis. It is actually required to prevent inappropriate spontaneous entry of cells into the cell cycle, resulting in hyperproliferation of activated and differentiated keratinocytes and apoptosis. Indeed, loss of the actin-severing functions carried out by ADF and CFL1 result in tissue breakdown, loss of barrier function, infection, and death (see mouse pathology later in Figure S7).

Loss of ADF/CFL1 Leads to Actin Filament Accumulation

We next addressed how the actin cytoskeleton was visibly perturbed in $ADF^{-/-}$ $CFL1^{-/-}$ skin keratinocytes that displayed a more-mesenchymal-like, elongated, and flattened morphology compared to their WT ($ADF^{+/+}$ $CFL1^{+/+}$) counterparts that have an epithelial morphology and grow in more-compact colonies (Figure 2A). When the ADF/CFL1-depleted cells were stained with phalloidin, we saw a huge increase in F-actin, in a form that appeared akin to stress fibers that are not typically abundant in epithelial cells. This was in stark contrast to WT keratinocytes, which displayed predominantly cortical actin (Figure 2A).

Because keratinocytes have a finite lifespan in culture, we therefore generated H-Ras-dependent malignant keratinocytes (squamous cell carcinoma [SCC] cells) after two-stage chemical carcinogenesis using DMBA/TPA (McLean et al., 2001; Quintanilla et al., 1986). We used $ADF^{-/-}$ $CFL1^{+/+}$ mice to generate SCCs (hereafter ADF-null SCCs) and knocked down CFL1 using siRNAs to generate SCC cells that were doubly depleted for ADF and CFL1. Specifically, pools of four siRNAs, as well as two individual siRNAs, were used, with the latter targeting either the coding region ($siCFL1^{CR}$) or the UTR ($siCFL1^{UTR}$) of CFL1 mRNA to control for off-target effects. The degree of CFL1 knockdown was excellent ($\geq$99% at the protein and mRNA level; Figure S1B and data not shown, respectively).

When ADF-null SCCs were depleted of CFL1 (siCFL1), the cells displayed altered morphology at 36–40 hr post-siRNA treatment, acquiring a flattened and more-spread phenotype with protrusions (Figure 2B). Similar to the $ADF^{-/-}$ $CFL1^{-/-}$ keratinocytes, when these cancer cells were stained with phalloidin, we observed a huge increase in F-actin in the form of cytoplasmic stress fibers in all the depleted cells (Figure 2D). This was observed in four different ADF-null SCC lines derived from separate animals, as well as with WT SCC cell lines co-transfected with siRNAs for both ADF and CFL1 (not shown). Enforced expression of WT CFL1 via retroviral infection of ADF-null SCCs (Figures S1C and S1D) prevented the accumulation of F-actin bundles and restored normal cell morphology when the endogenous protein was depleted by UTR-targeted siRNAs (Figure 2C). Comparing the relative transcription levels of ADF/cofilin family members in WT SCC cells, we found that CFL1 is the predominant isoform, with a 7- to 8-fold increase over ADF and ~35-fold increase over CFL2. ADF and CFL2 differed by ~4.5-fold (Figure S1E). Whereas we could restore cell morphology by modest re-expression of exogenous CFL1, we also found that enforced overexpression of CFL2 could also partially restore cell morphology and suppress F-actin accumulation upon ADF/CFL1 depletion (Figures S1F–S1H and S1J).

Because cofilins have reported roles in cell migration, membrane protrusions, and lamelipodia dynamics, we next addressed whether ADF- and CFL1-depleted SCCs were still able to generate dynamic peripheral membrane structures. We found that over a 30-min period, siCFL1-treated ADF-null SCCs retained the ability to generate dynamic membrane protrusions (some in the form of blebs), although these were less frequent, more persistent, and less dynamic (see Figure 2E, representative kymographs and kymograph profiles in Figures 2F and 2G, and quantifications in Figures 2H–2J; the montage in Figure 2E was generated from Movies S1 and S2). Taken together, our data demonstrate an unequivocal role for ADF and CFL1 in the dynamic regulation of actin stress fibers in normal and malignant keratinocytes, ensuring that they are turned over and not assembled/stabilized in an uncontrolled manner. Membrane structures can still be generated in the absence of ADF and CFL1, but these are less dynamic.

Loss of ADF and CFL1 Increases Free Actin Barbed Ends, Stress Fibers, and Enlarged Focal Adhesions

The accumulation of stable cytoplasmic filaments prompted us to examine G-/F-actin ratios in doubly ADF/CFL1-depleted cells. We performed a two-step lysis involving phalloidin-mediated F-actin stabilization and observed a large increase in F- compared to G-actin (Figure 3A). In addition, the ADF/CFL1-depleted SCCs exhibited an ~7- to 8-fold increase in free-barbed ends, indicative of increased actin polymerization (Figure 3B; quantified in Figure 3C). Anti-phospho-paxillin and anti-vinculin IF showed that ADF/CFL1-depleted SCCs had a greater number of larger, more brightly stained adhesions and that many of the

(C) ADF-null SCCs expressing empty vector (pWZL) or exogenously expressed CFL1 (pWZL+$CFL1^{wt}$) were treated with siNT or siRNAs targeting the UTR ($siCFL1^{UTR}$) of CFL1 mRNA. Following siRNA treatment, cells were stained for F-actin.

(D) Percentage of ADF-null SCCs displaying aberrant actin accumulation following CFL1 depletion. 790 control and 615 CFL1 (total) depleted cells were quantified in four independent experiments. Result presented as mean ± SEM. ***unpaired t test p value < 0.0001.

(E) ADF-null SCCs treated with siNT or siCFL1 for 48 hr were imaged every 30 s for 30 min. Representative montages of cells ruffling or blebbing their membranes are shown. White bars with arrowheads drawn perpendicular to membrane protrusions represent examples of areas for kymograph analysis. Movies of these cells are provided as Movies S1 and S2.

(F) Representative kymograph profiles from three siNT- and four siCFL1-treated cells. Profiles were plotted using normalized intensity values from 1-pixel-wide representative kymographs of membrane protrusions from siNT- and siCFL1-treated cells, composed parallel to protrusion direction over 30 min.

(G) Representative kymographs generated from cells shown in (E).

(H–J) Number of protrusions per 30 min, protrusion length, and time for a complete protrusion extension and retraction (protrusion persistence) are shown. Twenty cells with 83 and 20 cells with 34 protrusions were quantified following siNT or siCFL1 treatment, respectively (n = 4). Circles represent all individual values, and bars represent means ± SD; ***Mann-Whitney p value $\leq$ 0.0002.

The scale bars represent 125 μm and 20 μm for phase and IF images, respectively. Nuclei were counterstained with DAPI in IF experiments. See also Figures S1B–S1H and S1J.

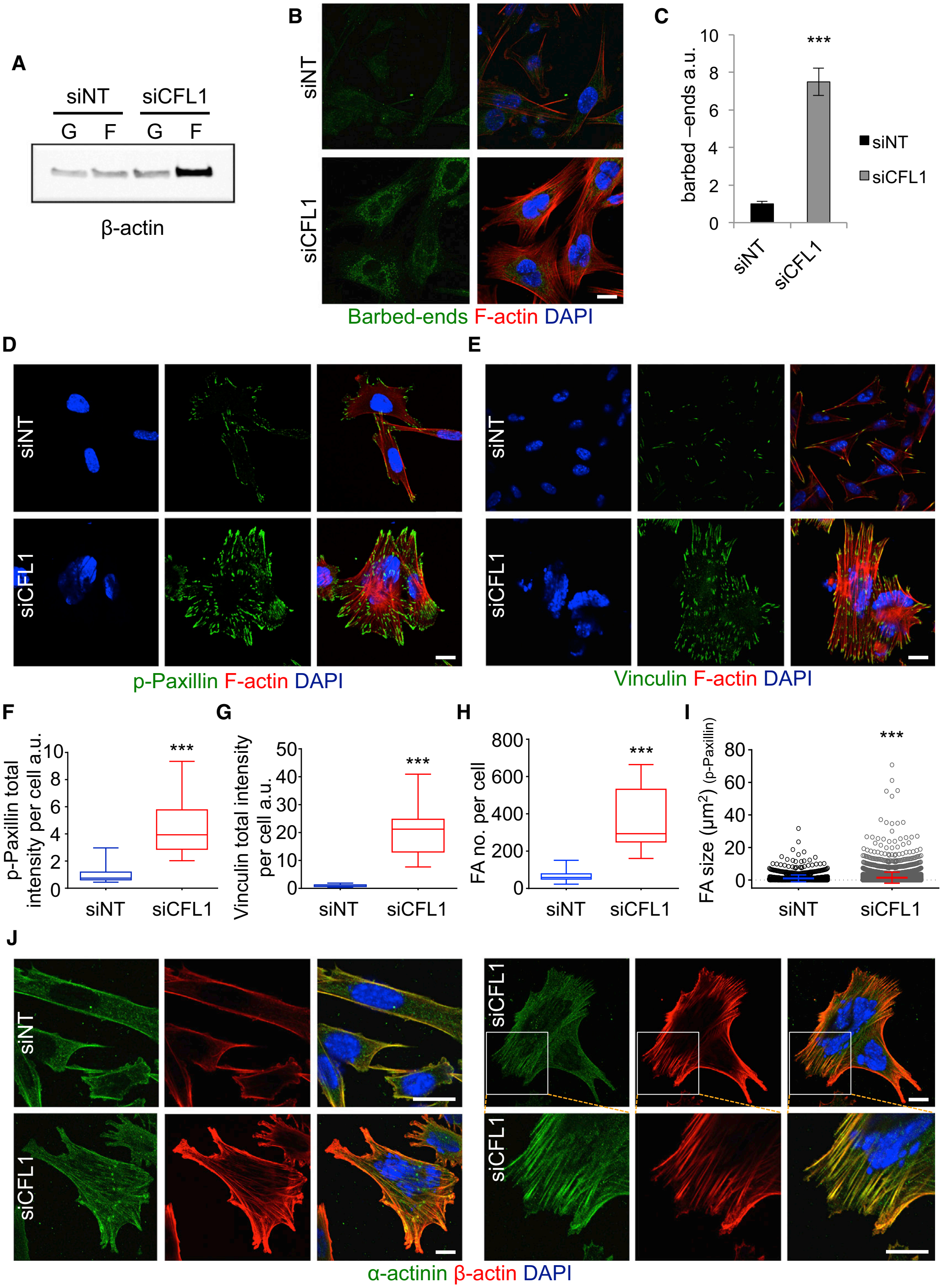

Figure 3. ADF/CFL1 Depletion Increases F-Actin Bundles and Focal Adhesions

(A) ADF-null SCCs were assessed by immunoblotting for globular (G) and filamentous (F) β-actin levels following treatment with siNT or siCFL1.

(B) Free barbed-end and F-actin fluorescent visualization of ADF-null SCCs treated as in (A).

(C) Quantification of barbed-end formation of ≥40 cells using ImageJ. Result presented as mean ± SEM (n = 2). ***unpaired t test p value < 0.0001.

(D and E) Focal adhesions were visualized with phospho(p)-paxillin (Tyr118) and vinculin after 48 hr of siRNA treatment. F-actin staining is also shown.

(legend continued on next page)

actin filaments were anchored into these at their plasma membrane ends (Figures 3D and 3E; quantified in Figures 3F–3I). We also examined whether the fibers accumulated upon ADF/CFL1 co-depletion are crosslinked. Staining for both α-actinin and β-actin revealed co-localization between the two proteins, indicating that the filaments are bundled (Figure 3J).

Actin Filament Accumulation Causes Loss of Nuclear Shape and Integrity

Our data imply that the elongated flattened morphology of the ADF/CFL1-depleted cells was the result of massive unrestrained accumulation of bundled actin filaments. We also observed profound nuclear deformation in both untransformed $ADF^{-/-}$ $CFL1^{-/-}$ keratinocytes and in ADF/CFL1-depleted SCC cells. Nuclei were either pushed into the bulk space available in between actin fibers or were compressed to cause loss of nuclear integrity via disruption of the nuclear envelope as judged by staining of Lamins A/C (see Figures 3D and 4A). A three-dimensional model compiled from z stacks of nuclear and F-actin-stained ADF/CFL1-depleted SCCs revealed that unrestrained actin filaments extend into, and through, nuclear space (Figure 4B). By contrast, the major actin filaments are around the cell periphery, with no nuclear encroachment, in ADF-null SCCs treated with non-targeting siRNAs (siNT) (Figure 4B). The same conclusions held true when cells were imaged with structured illumination microscopy (SIM) that permitted enhanced visualization of the relationship between actin filaments and the nuclear envelope, visualized by Lamin A/C staining (Figure 4C and z stack in Figure S3A).

To test whether the unregulated actin filaments were also associated with nuclear shape changes in vivo, $ADF^{-/-}$ $CFL1^{-/-}$ and control $ADF^{+/+}$ $CFL1^{+/+}$ mouse skins were stained for β-actin and Lamin A/C. Cells throughout the thickened epidermis in double ADF/CFL1 knockout mice contained vastly increased numbers of cytoplasmic actin filaments that were not visible in control mice and deformation of nuclear shape with disruption of nuclear Lamin A/C and DAPI staining (Figure 4D). Some nuclei were fragmented and showed a jumbled arrangement within the mutant tissue. There was general enlargement (karyomegaly; Figure 4E), suggestive of cellular stress (Chow et al., 2012), and there was breakdown of tissue architecture. A corresponding increase in cytoplasmic filamentous actin and aberrant nuclear morphology were also apparent when ADF and CFL1 were depleted in hepatocytes of the adult liver (Figure S3B). Taken together, our results so far suggested that accumulation of actin stress fibers triggered by ADF/CFL1 loss may physically promote deformation of the nuclear lamina—a hypothesis we test below.

We next interfered with known actin filament regulators. The major mechanisms by which actin filaments are generated within cells are 3-fold: spontaneous actin nucleation and elongation that depends on the actin monomers concentration; branched nucleation by the Arp2/3 complex; and, lastly, nucleation and elongation mediated by formins (reviewed in Pollard and Cooper, 2009). Therefore, we depleted Arp3 and the two major diaphanous-related formins mDia1 and mDia2 (siRNA-mediated knockdown is shown in Figures S4A and S4B). Although there were some mild morphological effects upon depleting mDia1 or mDia2 when compared to control ADF-null SCCs (Figure 5A), there was no rescue of the changes in flattened elongated cell morphology, actin stress fiber accumulation, or nuclear deformation phenotypes caused by ADF/CFL1 co-depletion (Figures 5A–5D). In contrast, knockdown of Arp3 caused reversion of the large flattened morphology, stress fiber production, and nuclear deformation (Figures 5A–5D). Although this effect of Arp3 depletion may not be direct, it visibly inhibits the extensive F-actin accumulation and nuclear deformation that results from ADF/CFL1 loss (Figures 5C and 5D).

In keeping with the siRNA-mediated knockdown experiments, the Arp2/3 inhibitor (CK869; Nolen et al., 2009) gave rise to a dose-dependent inhibition of F-actin that accumulated after ADF/CFL1 loss (Figures S4C and S4E), whereas the general "small-molecule inhibitor of formin homology 2 domain" (SMIFH2) (Rizvi et al., 2009) did not inhibit F-actin accumulation (Figures S4D and S4F).

We also considered whether SRF/MAL transcriptional activity, which is known to be enhanced when G-actin concentration is low (reviewed in Olson and Nordheim, 2010; Sotiropoulos et al., 1999; Vartiainen et al., 2007), contributed to the observed phenotypes, because a subset of genes regulated by their activity (including actin itself) was upregulated in ADF/CFL1-depleted cells (not shown). Therefore, we depleted SRF by RNAi in order to indirectly modulate actin accumulation in doubly ADF/CFL1-depleted cells (Figure S5A). We found that SRF depletion significantly restored a more-normal morphology and suppressed aberrant actin filament accumulation upon ADF/CFL1 depletion (Figures 5A, 5B, 5E, and S5B). This implied that SRF-mediated transcription was required to fuel the enhanced actin stress fiber assembly in the absence of cofilin-mediated dynamic actin filament turnover. Importantly, SRF depletion also significantly restored the number of non-fragmented nuclei (Figure 5C), providing supporting evidence that it is the over-assembly and stabilization of actin stress fibers upon loss of the filament-severing proteins ADF and CFL1 that causes nuclear deformation. Severalfold overexpression of CFL2 also led to reduction of F-actin accumulation as noted above, which, in turn, could significantly restore nuclear shape and lamina integrity after ADF/CFL1 loss (Figures S1H–S1J).

(F–H) Quantification of p-paxillin and vinculin staining, as well as the number of focal adhesions (FAs) per cell based on p-paxillin staining was performed using ImageJ. Box plots display data from min to max. Forty cells quantified for p-paxillin and 63 for vinculin staining in greater than or equal to three fields (n = 2) are shown. ***Mann-Whitney p value < 0.0001.

(I) Focal adhesion size, based on p-paxillin staining, of 1,590 focal adhesions from siNT- and >5,000 from siCFL1-treated cells was quantified in ImageJ. Circles represent all individual measurements, and bars present mean ± SD. ***Mann-Whitney p value < 0.0001.

(J) IF staining of siNT-/siCFL1-treated cells for α-actinin and β-actin. Two examples of siCFL1-treated cells are shown, and zoomed images of boxed region are shown on lower right panels.

The scale bars represent 20 μm.

OPEN ACCESS
CellPress

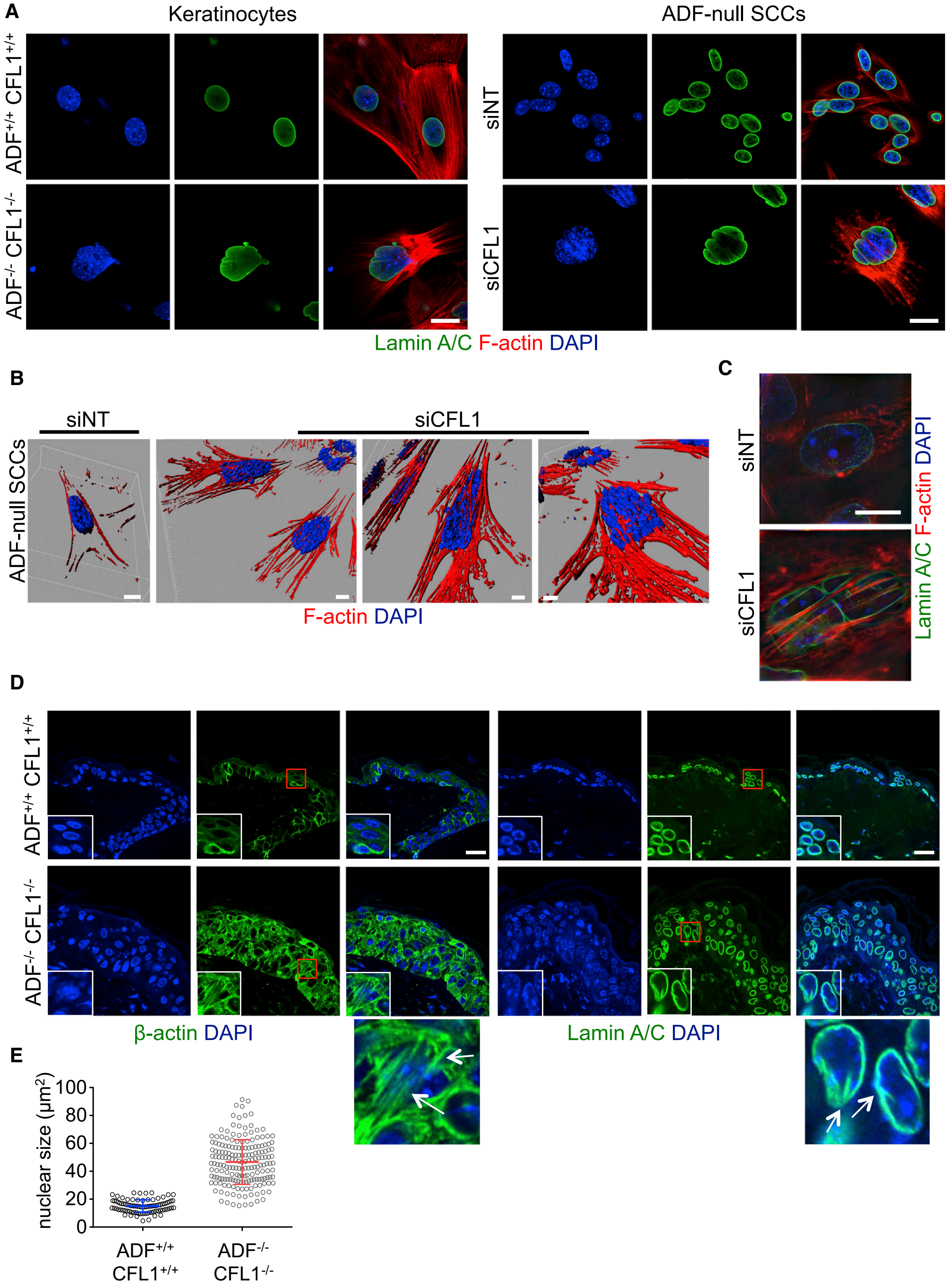

Figure 4. Actin Accumulation Causes Nuclear Deformation

(A) The nuclear envelope of keratinocytes (left panel) and siNT- or siCFL1-treated ADF-null SCCs (right panel) was stained for Lamin A/C. The scale bar represents 20 μm.

(legend continued on next page)

Actomyosin Contractility Is a Critical Mediator of Nuclear Deformation

We next investigated whether the accumulated actin filaments were decorated with phospho-myosin, as an indicator of contractility. Control cells displayed only peripheral staining of phosphorylated (Ser19) myosin light chain (pMLC) that was coincident with cortical actin filaments, whereas the actin bundles crossing the intracellular space stained heavily for pMLC in the ADF/CFL1-depleted cells (Figure 6A). This implied that the induced actin filaments were contractile, resulting in increased intracellular tension in the absence of ADF/CFL1.

We next used inhibitors of both Rho-associated protein kinase (ROCK; Y27632) and myosin II activity (Blebbistatin) (Kovács et al., 2004; Straight et al., 2003; Uehata et al., 1997). There was some retraction of cell bodies when these inhibitors were used in control siNT-treated cells, with the appearance of some thin membrane protrusions (Figure 6B). However, when Blebbistatin was used in ADF/CFL1 co-depleted cells, there was partial restoration of normal cell morphology (Figure 6B), suppression of stress fiber accumulation (Figure 6B), and rescue of the nuclear deformation phenotype as judged by a significantly increased number of non-fragmented nuclei (Figure 6C). In contrast, the ROCK inhibitor Y27632 did not rescue the nuclear deformation phenotype. We also found that Myosin IIA, the main non-muscle myosin isoform expressed in SCCs (data not shown), decorates the filaments induced upon ADF/CFL1 loss (Figure 6D). siRNA-mediated depletion of Myosin IIA efficiently prevented actin accumulation and rescued nuclear fragmentation after ADF/CFL1 co-depletion (Figures 6E–6G).

Nuclear Deformation Requires the LINC Complex

We next addressed whether the physical damage to the nucleus caused by the accumulated contractile stress fibers involved the linker of cytoskeleton to nucleoskeleton (LINC) complex, which provides a physical link between the actin cytoskeleton and nuclear lamina (Crisp et al., 2006; Zhen et al., 2002; reviewed in Starr and Fridolfsson, 2010). Therefore, we depleted two of the components of the LINC complex, namely Nesprin-2 giant (hereafter Nesprin-2G; also known as Syne-2) and SUN1 (Figure 7). Disruption of the LINC complex is known to affect nuclear shape in epidermal cells (Lüke et al., 2008), and this was the case when Nesprin-2G was depleted in control cells (siNT; Figure 7A, arrows in top right panels; see Figure 7B for knockdown efficiency [determined by qRT-PCR in this case, as we did not have a reliable antibody]). However, when Nesprin-2G was knocked down in ADF/CFL1-depleted cells, nuclear shape was restored and was similar to Nesprin-2G knockdown in control cells (compare lower right to upper right panels in Figure 7A). Nesprin-2G-depleted cells exhibited a less-flattened morphology with slightly enlarged nuclei visibly projecting away from the cell surface (Figure 7C) and reduced nuclear deformation (Figure 7D). Upon co-staining of the nuclear envelope and F-actin (see Figure 7A, lower panels), we observed an "actin gap" that formed around the nucleus when Nesprin-2G was knocked down in ADF/CFL1 co-depleted cells. When we quantified this actin gap, i.e. space around the nucleus representing the distance between the nuclear envelope and the closest actin filaments, we found a significant increase upon Nesprin-2G knockdown, without overall cellular filamentous actin levels being significantly altered (Figures 7E and 7F). This likely reflects uncoupling of the accumulated actin filaments from the nuclear envelope caused by disruption of the LINC complex. In such a scenario, the nucleus would be freed from the increased tension and excessive force produced by the contractile actin stress fibers linking to the nucleus via the LINC complex upon loss of both ADF and CFL1, so preventing nuclear deformation. Similar effects and rescue of nuclear fragmentation was observed upon SUN1 co-depletion (Figures 7G–7I), indicating that the mechanism by which the uncontrolled actin stress fibers damage the nuclear lamina involves engagement with the LINC complex.

ADF/CFL1 Depletion Ultimately Leads to DNA Damage and Cell Death

Because the ultimate consequence of these events was loss of cell viability in vivo (Figures 1G and S7), we investigated the fate of cells depleted of ADF/CFL1 in vitro. Proliferation halted 24 hr after siCFL1 treatment, and almost the entire cell population shifted to a sub-G1 peak by 72 hr when compared to the siNT control (Figure S6A), indicative of massive apoptosis. We found that several apoptosis markers, including cleaved caspases and lamin A, were elevated by 48 hr, which paralleled the timing of complete loss of CFL1 in ADF-null SCCs (Figure S6B). Moreover, Plk-1 and full-length PARP levels were decreased by 72 hr, indicative of cell cycle exit and apoptosis, respectively. There was also evidence of a DNA damage response (γH2AX blot in Figure S6B), backed up by staining of cells and skin for the double-strand break marker γH2AX (Figures S6C–S6E). We note that the LINC complex is known to be associated directly or indirectly with chromatin, through binding to nuclear lamina. Therefore, it is intriguing to speculate that the nuclear deformation we describe upon ADF/CFL1 co-depletion may be directly linked to DNA damage; however, we have not been able to discriminate between damage caused due to nuclear membrane deformation/rupture or as a result of induction

(B) 3D model construction composed of nuclei (blue) and F-actin (red) of cells treated as in (A). Model constructed using IMARIS software (Bitplane) after 0.4-μm optical slices were obtained using confocal microscopy. The siNT scale bar represents 7 μm and siCFL1 scale bars represent 10 μm, 20 μm, and 10 μm (left to right images).

(C) Images of ADF-null SCCs treated with siNT or siCFL1 were obtained by structured illumination microscopy (SIM). Cells were stained for Lamin A/C. Images were taken as part of a z stack, available as Figure S3A. The scale bar represents 10 μm.

(D) IF staining for β-actin (left panels) and Lamin A/C (right panels) performed on skin sections. Zoomed images of boxed region are shown below panels. The scale bars represent 20 μm.

(E) Nuclear size quantification based on Lamin A/C staining of 88 control and 183 double knockout nuclei (n = 2) performed in ImageJ. All individual measurements are presented, and bars represent mean ± SD. ***Mann-Whitney p value < 0.0001.

Nuclei and F-actin were counterstained with DAPI and phalloidin, respectively. See also Figure S3.

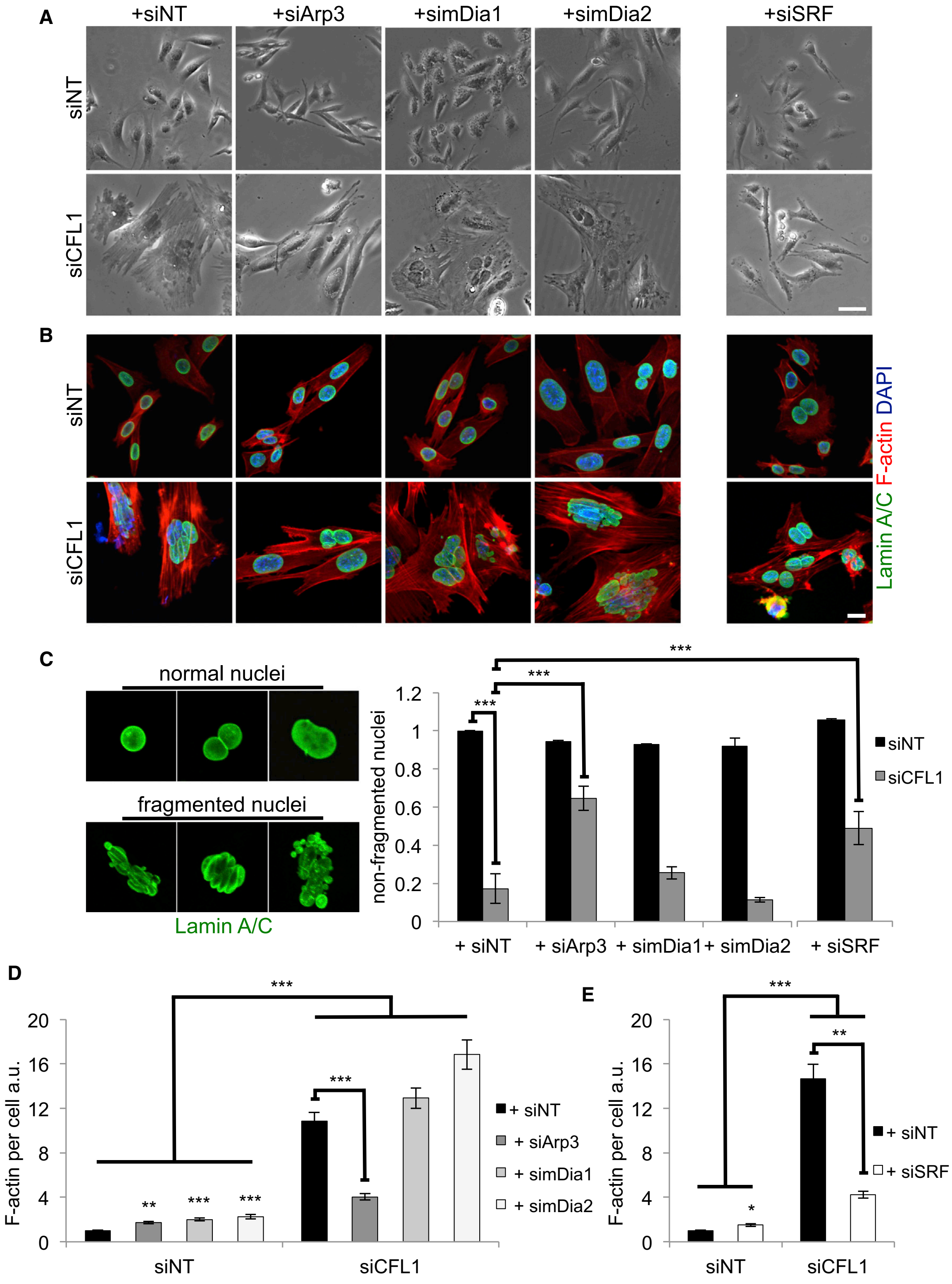

Figure 5. Modulating Actin Filaments by Suppressing Arp3 and SRF Rescues Nuclear Deformation

(A) Phase contrast images from ADF-null SCCs treated with siNT or siCFL1 alone or together with siRNAs for Arp3, mDia1, mDia2, or SRF, respectively.

(B) IF staining of cells after the treatments above for Lamin A/C.

(legend continued on next page)

of apoptosis. Adult mouse liver depleted for ADF and CFL1 also exhibited evidence of massive accumulation of F-actin and associated DNA damage, with a significant proportion of hepatocytes staining positively for γH2AX (Figures S6F and S6G).

To understand the long-term consequences of deleting the genes encoding ADF and CFL1 for the animal, samples from the organs of sacrificed ADF$^{-/-}$ CFL1$^{-/-}$ mice 4 days after treatment with 4-OHT were taken for pathological examination. Major lesions were found in the skin, conjunctiva, cornea, and oral mucosa. Pronounced histological changes were noted, with diffuse thickening of the epidermis due to hyperplasia of the spinous layer of cells, which had enlarged misshapen and disorganized nuclei (Figure S7A, upper left panel, solid arrow). Within both the underlying basal and spinous cell layers, numerous apoptotic cells with condensation, hypereosinophilia, and nuclear pyknosis were evident (Figure S7A, upper right panel). Histological changes were also evident in the cornea, which displayed acute keratitis with diffuse stromal edema, neutrophil infiltrates, misshapen nuclei, and apoptosis of superficial stratified epithelial cells and frank ulceration (Figure S7B, solid arrow). Finally, similar changes were evident in the buccal and lingual mucosae in the oral cavity (Figure S7C). This demonstrates that severe disruption of tissue homeostasis is evident upon pathological examination, and this is associated with perturbed nuclear shape and apoptosis in vivo.

DISCUSSION

Double deletion of ADF and CFL1 in keratinocytes and hepatocytes in vivo had the profound effects we describe, induced as soon as could be determined after ADF/CFL1 were co-depleted, highlighting their direct importance in cellular health and tissue homeostasis. Essentially, ADF and CFL1 are required to (1) prevent inappropriate spontaneous proliferation of keratinocytes that express differentiation markers, (2) maintain cell-cell contacts and membrane E-cadherin, (3) suppress keratinocyte "activation," (4) prevent over-accumulation of contractile actin stress fibers and associated focal adhesions, and (5) prevent nuclear deformation and ultimately apoptosis/DNA damage. The hyperproliferative phenotype may be a consequence of loss of cell-cell contacts that has previously been linked to release from a non-proliferating state (Charest et al., 2009; Owens et al., 2000). Mechanical force, derived from the dramatic overproduction of contractile actin stress fibers, may also contribute. One possibility we are investigating is the role of YAP/TAZ signaling in keratinocyte hyperproliferation, as this is controlled by actin-regulatory proteins, including CFL1, and mechanically induced proliferation (Aragona et al., 2013), and we have found that YAP/TAZ target genes are increased in doubly ADF- and CFL1-depleted SCC cells (not shown).

Keratinocyte activation and differentiation are known to be influenced by cell shape changes. For example, actin networks have been linked to keratinocyte differentiation via elevated SRF/MAL signaling (Connelly et al., 2010). We found that double depletion of ADF and CFL1 in SCC cells promoted expression of SRF/MAL transcriptional targets (not shown), including a predominance of cytoskeletal, cell adhesion, and actin regulatory molecules (Olson and Nordheim, 2010), and that SRF knockdown led to decreased actin accumulation and significant rescue of nuclear deformation. This implied that lack of actin severing, via depletion of ADF/CFL1, promoted unrestrained incorporation of G-actin into actin filaments (F-actin), which, in turn, led to a very low G-:F-actin ratio. As a result, compensatory SRF/MAL transcriptional activity was triggered, as has been described by others (Miralles et al., 2003; Sotiropoulos et al., 1999), fueling the production of even more actin filaments that contributes to the severe consequences observed.

Live-cell imaging established that peripheral membrane protrusions, in the SCC cells examined, were able to form but that their persistence and dynamics were influenced by loss of ADF and CFL1. This is in line with reports showing that CFL1 mediates treadmilling activities that primarily control sub-cortical dendritic actin networks, promoting membrane protrusion and lamellipodia upon receipt of appropriate stimuli (Bravo-Cordero et al., 2013; Delorme et al., 2007; Ghosh et al., 2004; Mouneimne et al., 2006; Pollard and Borisy, 2003). Unexpectedly, our data indicate that one important and redundant role of the actin-filament-severing functions of ADF and CFL1 is to turnover cytoplasmic contractile actin stress fibers, so promoting their dynamic regulation, keeping them under tight control. The severing activity of cofilin(s) is concentration dependent (Andrianantoandro and Pollard, 2006), and CFL2, which has much-weaker actin-severing activity than ADF and CFL1 (Vartiainen et al., 2002), cannot compensate for loss of ADF and CFL1 in skin (or liver) or SCCs at endogenous levels and its expression is not altered upon ADF/CFL1 loss. However, when overexpressed several fold in cancer cells, it is able to prevent aberrant actin fiber formation and nuclear deformation to some extent. Hence, it is likely that the difference in severing ability between CFL1 and CFL2 explains the lack of compensation in dynamically regulating actin stress fibers.

The unrestrained accumulation of contractile stress fibers in keratinocytes had unrecoverable consequences for viability. There was profound nuclear deformation and disruption to the nuclear membrane, leading to apoptosis and DNA damage in vitro and in vivo. In some cases, this led to leakage of nuclear material, including the transcription factors c-Myc and SRF (not shown). Interference with Arp3, but not mDia1 and mDia2, inhibited actin stress fiber and nuclear deformation phenotypes

(C) (Left) Representative images of nuclei classified as having normal morphology or being deformed/fragmented. Staining is for Lamin A/C. (Right) Quantification of cells harboring non-fragmented nuclei following the siRNA treatments described is shown. Results are presented as mean ± SEM (n = 3). Greater than 150 cells were counted per condition in every repeat, contained in greater than or equal to three fields. ***one-way ANOVA with Tukey's multiple comparison test; p value $\leq$ 0.0005.

(D and E) Quantification of F-actin levels per cell performed in ImageJ. Greater than or equal to 95 cells were quantified per condition (n = 3) for Arp3 and mDias, and between 30 and 81 cells were quantified per treatment (n = 2) for SRF depletion experiments, accordingly. Data are represented as mean ± SEM. Kruskal-Wallis ANOVA with Dunn's multiple comparison test; *p value $\leq$ 0.05; **p value $\leq$ 0.01; ***p value $\leq$ 0.0001.

The scale bars represent 60 μm for phase and 20 μm for IF images. See also Figures S1H–S1J, S4, and S5.

OPEN
ACCESS
CellPress

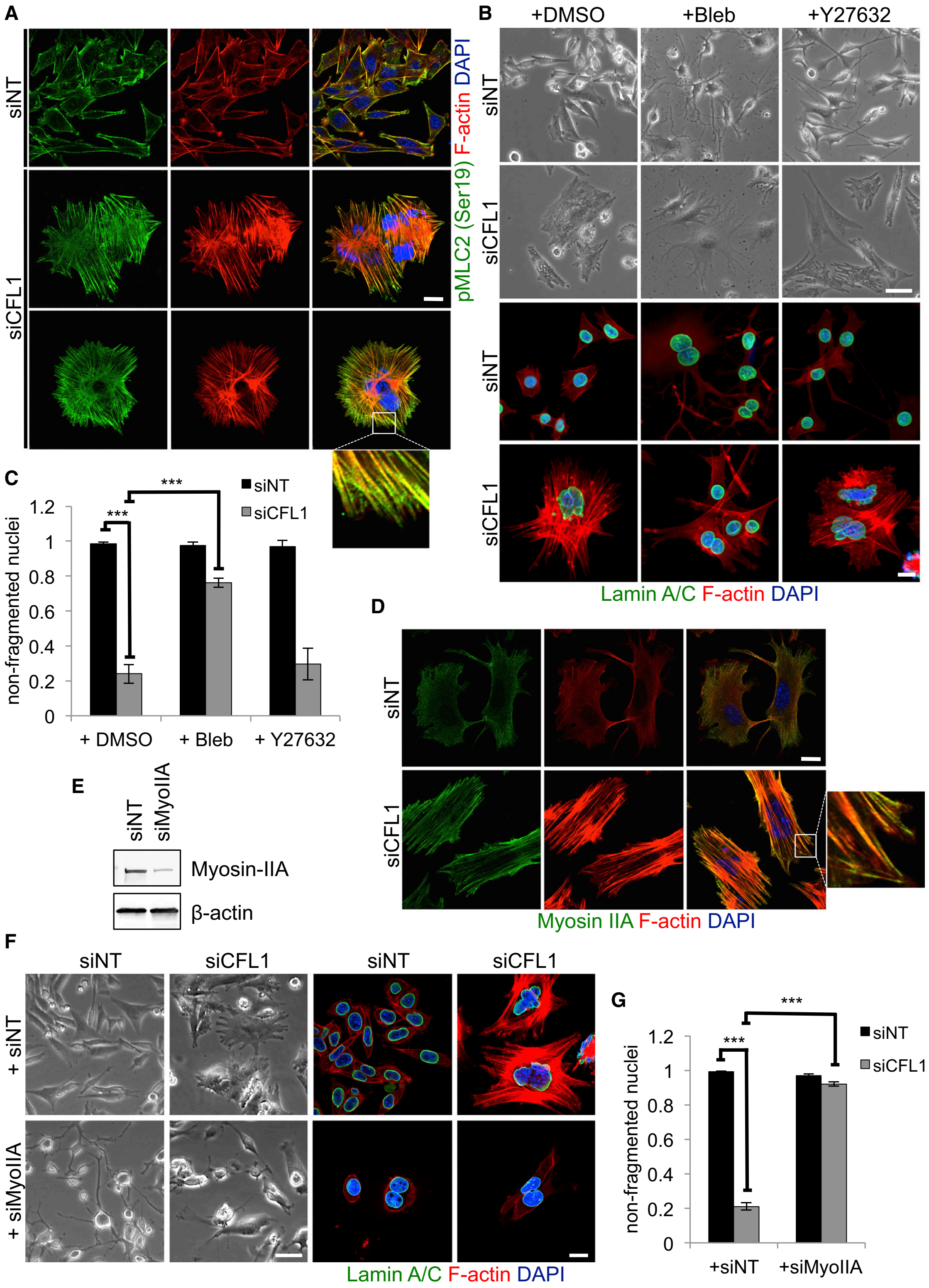

Figure 6. Enhanced Contractility Contributes to Nuclear Deformation in Cells Lacking ADF/CFL1

(A) IF staining for phospho-MLC of ADF-null SCCs treated with siNT or siCFL1.

(B) Phase contrast images of cells treated as in (A) and incubated overnight with DMSO, 25 μM Blebbistatin, or 20 μM Y27632 (top panels). Cells were also stained for Lamin A/C (lower panels).

(legend continued on next page)

upon ADF/CFL1 depletion. Whereas Arp2/3's role in the unrestrained actin stress fiber accumulation may be via indirect effects on cytoskeletal networks, the fact that nuclear deformation is also rescued when Arp3 is depleted implies that the aberrant actin filaments are likely responsible. Whereas the inability of knockdown of mDia1 or mDia2 to suppress actin fiber accumulation, or the use of the SMIFH2 inhibitor, do not exclude the possibility that other formins may play a role in the stress fiber accumulation, the lack of effect on aberrant stress fibers correlated with lack of any effect on nuclear deformation. It was the case that aberrant accumulation of actin stress fibers was always associated with nuclear deformation and disruption of the nuclear lamina.

The uncontrolled bundled actin filaments promoted by ADF/CFL1 depletion were decorated with pMLC, implying their contractile nature, and we note that a direct role of ADF and CFL1 in regulating intracellular contractility in human cells has been proposed (Wiggan et al., 2012). Cofilins are thought to compete with myosin for binding to actin filaments, thereby inhibiting excessive contractility and force generation inside cells. Therefore, the absence of ADF/CFL1 could lead to unrestricted myosin binding to the new filaments that exert excessive contractile force this way. We established that contractility was causally linked to the extensive nuclear deformation observed upon ADF/CFL1 co-depletion, because inhibition of myosin activity by Blebbistatin or Myosin-IIA knockdown efficiently inhibited actin stress fiber accumulation and the nuclear deformation they cause.

One way that cells can regulate their nuclear shape is via an actin cap—a subset of contractile actin filaments passing over the nucleus and terminating in distinct actin-cap-associated focal adhesions (Khatau et al., 2009; Kim et al., 2012). It was recently reported that increased tension on the actin-cap-linked stress fibers promotes the formation of indentation sites in the nuclear lamina that also affect chromatin condensation (Versaevel et al., 2014). The phospho-myosin-containing actin cap fibers are physically connected to the nuclear lamina by the so-called LINC complex (Khatau et al., 2009; Starr and Fridolfsson, 2010). This raised the question of whether disruption of the nuclear lamina upon ADF and CFL1 depletion involved the LINC complex. We therefore depleted Nesprin-2 giant and SUN1, components of the LINC complex that tether actin filaments from the cytoplasm to the nuclear membrane (Crisp et al., 2006). This resulted in a decoupling of the actin cytoskeleton from the nuclei upon knockdown of Nesprin-2G, or SUN1 knockdown, and this rescued the nuclear deformation phenotype induced by ADF/CFL1 co-depletion.

In addition to the phenotypes we describe, double depletion of ADF and CFL1 commonly resulted in multi-nucleation. We have not focused on this aspect of the phenotype here as it has been reported previously in other systems and it is likely due to impaired final stages of cell division caused by dysfunctional contractile rings (Hotulainen et al., 2005; Tahtamouni et al., 2013).

Taken together, our results demonstrate that the uncontrolled contractile actin stress fibers formed upon co-depletion of ADF and CFL1 are responsible for promoting nuclear deformation, likely by exerting increased contractile force to the nuclear envelope via its attachments to the LINC complex. This implies that dynamic regulation of actin stress fibers by cofilins is vital for the actin-LINC complex to control nuclear shape, movement, and integrity that is lost in an array of diseases, collectively termed as laminopathies (Dauer and Worman, 2009; Gundersen and Worman, 2013; Stewart et al., 2007).

EXPERIMENTAL PROCEDURES

Experiments involving animals were carried out in accordance with the UKCCCR guidelines by approved protocol (HO PL 60/4248). Brief experimental procedures are listed here. For details, please see the Supplemental Experimental Procedures.

Generation of Transgenic Mice

Generation of transgenic mice with either deletion of ADF (Bellenchi et al., 2007), targeted loxP sites in the *CFL1* gene (Gurniak et al., 2005), or expressing the modified Cre recombinase-estrogen receptor fusion under control of the Keratin-14 promoter (K14CreER^{T2}; Li et al., 2000) has been described. To enable cell-type-specific CFL1 ablation, ADF$^{-/-}$ mice and CFL1$^{fl/fl}$ mice were mated to K14CreER^{T2} mice. This resulted in offspring that carried K14CreER^{T2} and were nullizygous for ADF and homozygous for the floxed *CFL1* gene (K14CreER^{T2}/ADF$^{-/-}$/CFL1$^{fl/fl}$), offspring that were nullizygous for ADF but carried two copies of the WT *CFL1* allele (K14CreER^{T2}/ADF$^{-/-}$/CFL1$^{WT/WT}$) or offspring that carried two copies of the WT *ADF* allele but were homozygous for the floxed *CFL1* gene (K14CreER^{T2}/ADF$^{WT/WT}$/CFL1$^{fl/fl}$).

Preparation and Administration of 4-OHT

Tamoxifen (4-OHT) was administered to 60-day-old K14-ADF mice as described (Indra et al., 1999) with minor modifications. The animals were then left for 10 days before experiments initiated except for K14CreER^{T2}/ADF$^{-/-}$ CFL1$^{fl/fl}$ mice, which were culled and tissue collected at 4 or 5 days. For in vitro experiments, 4-OHT was dissolved in ethanol and used at a final concentration of 10 nM.

Isolation of Primary Keratinocytes from Mouse Tails

Tail skin was collected from mice with the desired genotype, and epidermis was removed and processed to dissociate keratinocytes that were maintained on collagen-I-coated plates.

Generation of SCC Cell Lines

Ras-driven chemically induced tumors were introduced to mice as described (McLean et al., 2001). Tumors derived from mice nullizygous to ADF were utilized to generate ADF-null SCC cell lines.

(C) Quantification of cells that, after these treatments, showed non-fragmented nuclei. Results are presented as mean ± SEM (n = 3). Greater than 150 cells were counted per condition per repeat. ***one-way ANOVA with Tukey's multiple comparison test; p value $\leq$ 0.0001.
(D) IF staining of siNT-/siCFL1-treated cells for Myosin IIA.
(E) Knockdown efficiency of siRNAs targeting Myosin IIA (64 nM;siMyoIIA) was tested by immunoblotting.
(F and G) Phase contrast (left panels) and IF (right panels) images of ADF-null SCCs treated with siNT or siCFL1 in the absence or presence of siMyoIIA (F) and quantification of cells with non-fragmented nuclei following these treatments (G). Results are presented as mean ± SEM (n = 3). Greater than 150 cells were counted per treatment per repeat. ***unpaired t test; p value < 0.0001.
The scale bars represent 60 μm for phase and 20 μm for IF images.

OPEN
ACCESS
CellPress

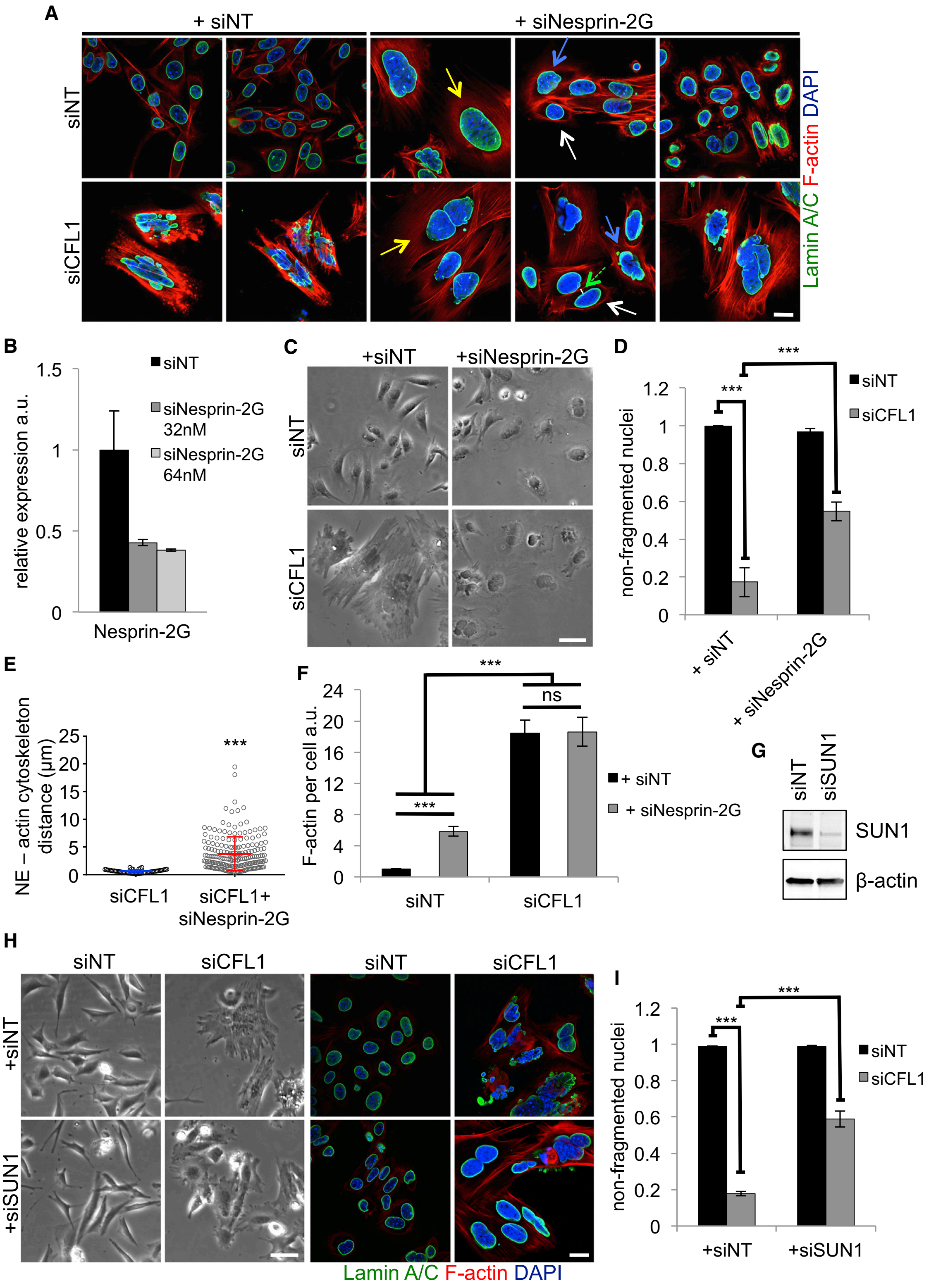

Figure 7. Uncoupling Actin Cytoskeleton from the LINC Complex Rescues Nuclear Deformation

(A) IF staining for Lamin A/C of ADF-null SCCs treated with siNT or siCFL1 alone or in conjunction with siRNAs targeting Nesprin-2G. White arrows designate normal nuclear morphology, blue arrows moderately misshaped, and yellow arrows enlarged nuclei. Green dashed arrow indicates a gap (white bar) between the nuclear envelope (NE) and the closest actin fibers.

(legend continued on next page)

CFL1 Cloning and Overexpression

CFL1 cDNA was cloned into the pWZL-Hygro retroviral vector. pEco packaging cells were used for virus production, and SCC cells were infected with addition of polybrene. Transformed cells were selected using hygromycin.

Free Barbed-Ends Assay

Free barbed-end formation was assessed and quantified by an adaptation of the method described previously (Worth et al., 2010).

Live-Cell Imaging, Kymograph, and Membrane Dynamics Analysis

Cells were imaged post-siRNA treatment using Scan^R screening station. Kymograph and membrane analysis were performed as previously described (Benjamin et al., 2010).

Inhibitor Treatments

Cells were transfected with siRNAs, treated with inhibitors 30 hr later, and incubated overnight before fixation.

SUPPLEMENTAL INFORMATION

Supplemental Information includes Supplemental Experimental Procedures, seven figures, one table, and two movies and can be found with this article online at http://dx.doi.org/10.1016/j.celrep.2015.10.056.

AUTHOR CONTRIBUTIONS

G.K., J.Z., and H.P. designed, performed, analyzed, and interpreted experiments in skin systems. J.Z. and V.G.B. analyzed the mouse skin phenotypes and characterized the keratin, E-cadherin, and cleaved caspase-3 expression in the mutant epidermis. R.A.R. and D.H. performed experiments in liver. C.B.G. provided key mouse strains and antibody reagents. E.S. and N.O.C. provided important advice. G.K., H.P., M.C.F., V.G.B., O.J.S., and W.W. designed and supervised the study. G.K. and M.C.F. co-wrote the manuscript with help from V.G.B. and O.J.S. All authors read the manuscript and approved its content prior to final submission.

ACKNOWLEDGMENTS

We thank Morwenna Muir for help with biological experimentation. This work was funded by Cancer Research UK (CRUK) Program Grant to M.C.F. and V.G.B. (no. C157/A15703) and European Research Council Advanced Investigator grant to M.C.F. (no. 294440 Cancer Innovation; supporting H.P.). It was also supported by CRUK China Fellowship to J.Z. (no. C157/A11682), by CRUK PhD studentship to G.K., by CRUK Beatson core program to O.J.S., and by AICR grant to M.C.F. (no. 09-0316) that funded H.P.

Received: June 11, 2015
Revised: August 21, 2015
Accepted: October 16, 2015
Published: November 19, 2015

REFERENCES

Andrianantoandro, E., and Pollard, T.D. (2006). Mechanism of actin filament turnover by severing and nucleation at different concentrations of ADF/cofilin. Mol. Cell *24*, 13–23.

Aragona, M., Panciera, T., Manfrin, A., Giulitti, S., Michielin, F., Elvassore, N., Dupont, S., and Piccolo, S. (2013). A mechanical checkpoint controls multicellular growth through YAP/TAZ regulation by actin-processing factors. Cell *154*, 1047–1059.

Bellenchi, G.C., Gurniak, C.B., Perlas, E., Middei, S., Ammassari-Teule, M., and Witke, W. (2007). N-cofilin is associated with neuronal migration disorders and cell cycle control in the cerebral cortex. Genes Dev. *21*, 2347–2357.

Benjamin, J.M., Kwiatkowski, A.V., Yang, C., Korobova, F., Pokutta, S., Svitkina, T., Weis, W.I., and Nelson, W.J. (2010). AlphaE-catenin regulates actin dynamics independently of cadherin-mediated cell-cell adhesion. J. Cell Biol. *189*, 339–352.

Bravo-Cordero, J.J., Magalhaes, M.A., Eddy, R.J., Hodgson, L., and Condeelis, J. (2013). Functions of cofilin in cell locomotion and invasion. Nat. Rev. Mol. Cell Biol. *14*, 405–415.

Bugyi, B., and Carlier, M.F. (2010). Control of actin filament treadmilling in cell motility. Annu. Rev. Biophys. *39*, 449–470.

Charest, J.L., Jennings, J.M., King, W.P., Kowalczyk, A.P., and García, A.J. (2009). Cadherin-mediated cell-cell contact regulates keratinocyte differentiation. J. Invest. Dermatol. *129*, 564–572.

Chow, K.H., Factor, R.E., and Ullman, K.S. (2012). The nuclear envelope environment and its cancer connections. Nat. Rev. Cancer *12*, 196–209.

Connelly, J.T., Gautrot, J.E., Trappmann, B., Tan, D.W., Donati, G., Huck, W.T., and Watt, F.M. (2010). Actin and serum response factor transduce physical cues from the microenvironment to regulate epidermal stem cell fate decisions. Nat. Cell Biol. *12*, 711–718.

Crisp, M., Liu, Q., Roux, K., Rattner, J.B., Shanahan, C., Burke, B., Stahl, P.D., and Hodzic, D. (2006). Coupling of the nucleus and cytoplasm: role of the LINC complex. J. Cell Biol. *172*, 41–53.

Dauer, W.T., and Worman, H.J. (2009). The nuclear envelope as a signaling node in development and disease. Dev. Cell *17*, 626–638.

Delorme, V., Machacek, M., DerMardirossian, C., Anderson, K.L., Wittmann, T., Hanein, D., Waterman-Storer, C., Danuser, G., and Bokoch, G.M. (2007). Cofilin activity downstream of Pak1 regulates cell protrusion efficiency by organizing lamellipodium and lamella actin networks. Dev. Cell *13*, 646–662.

(B) qPCR showing knockdown efficiency of siRNAs targeting Nesprin-2G. The data were normalized against cells treated with 64 nM siNT and are presented as mean ± SD. The primers are shown in Table S1.
(C) Phase contrast images of cells treated as in (A) (note that images of cells treated with siNT or siCFL1 [left panels] are the same as in Figure 5A, as they were part of the same experiment).
(D) Quantification of cells that displayed non-fragmented nuclei. Results are presented as mean ± SEM (n = 3). Greater than 150 cells were counted per condition per repeat. ***one-way ANOVA with Tukey's multiple comparison test; p value $\leq$ 0.0001.
(E) Quantification of the distance between the NE and the closest visible actin fibers in cells treated with siCFL1 alone or together with siNesprin-2G (see green dashed arrow in A). Thirty-six cells were quantified from greater than or equal to nine fields of each treatment (n = 2). Measurements were performed in ImageJ using images acquired with confocal microscopy; they were evenly spaced around the periphery of the nuclei and plot displays all individual measurements (distance in μm) ± SD. ***Mann-Whitney p value < 0.0001.
(F) Quantification of F-actin levels. Greater than or equal to 60 cells were quantified per condition (n = 3). Data are presented as mean ± SEM. ***Kruskal-Wallis ANOVA with Dunn's multiple comparison test; p value $\leq$ 0.0001.
(G) Knockdown efficiency of siRNAs targeting SUN1 (64 nM; siSUN1) was tested by immunoblotting.
(H and I) Phase contrast (left panels) and IF (right panels) images of ADF-null SCCs treated with siNT or siCFL1 alone or together with siSUN1 (H) and quantification of cells with non-fragmented nuclei following these interventions (I). Data are presented as mean ± SEM (n = 3). Greater than 150 cells were counted per treatment per repeat. ***unpaired t test; p value < 0.0001.
The scale bars represent 60 μm for phase and 20 μm for IF images. Nuclei and F-actin were counterstained with DAPI and phalloidin, respectively.

DesMarais, V., Ghosh, M., Eddy, R., and Condeelis, J. (2005). Cofilin takes the lead. J. Cell Sci. *118*, 19–26.

Freedberg, I.M., Tomic-Canic, M., Komine, M., and Blumenberg, M. (2001). Keratins and the keratinocyte activation cycle. J. Invest. Dermatol. *116*, 633–640.

Ghosh, M., Song, X., Mouneimne, G., Sidani, M., Lawrence, D.S., and Condeelis, J.S. (2004). Cofilin promotes actin polymerization and defines the direction of cell motility. Science *304*, 743–746.

Gundersen, G.G., and Worman, H.J. (2013). Nuclear positioning. Cell *152*, 1376–1389.

Gurniak, C.B., Perlas, E., and Witke, W. (2005). The actin depolymerizing factor n-cofilin is essential for neural tube morphogenesis and neural crest cell migration. Dev. Biol. *278*, 231–241.

Hotulainen, P., Paunola, E., Vartiainen, M.K., and Lappalainen, P. (2005). Actin-depolymerizing factor and cofilin-1 play overlapping roles in promoting rapid F-actin depolymerization in mammalian nonmuscle cells. Mol. Biol. Cell *16*, 649–664.

Ikeda, S., Cunningham, L.A., Boggess, D., Hawes, N., Hobson, C.D., Sundberg, J.P., Naggert, J.K., Smith, R.S., and Nishina, P.M. (2003). Aberrant actin cytoskeleton leads to accelerated proliferation of corneal epithelial cells in mice deficient for destrin (actin depolymerizing factor). Hum. Mol. Genet. *12*, 1029–1037.

Indra, A.K., Warot, X., Brocard, J., Bornert, J.M., Xiao, J.H., Chambon, P., and Metzger, D. (1999). Temporally-controlled site-specific mutagenesis in the basal layer of the epidermis: comparison of the recombinase activity of the tamoxifen-inducible Cre-ER(T) and Cre-ER(T2) recombinases. Nucleic Acids Res. *27*, 4324–4327.

Khatau, S.B., Hale, C.M., Stewart-Hutchinson, P.J., Patel, M.S., Stewart, C.L., Searson, P.C., Hodzic, D., and Wirtz, D. (2009). A perinuclear actin cap regulates nuclear shape. Proc. Natl. Acad. Sci. USA *106*, 19017–19022.

Kim, D.H., Khatau, S.B., Feng, Y., Walcott, S., Sun, S.X., Longmore, G.D., and Wirtz, D. (2012). Actin cap associated focal adhesions and their distinct role in cellular mechanosensing. Sci. Rep. *2*, 555.

Kovács, M., Tóth, J., Hetényi, C., Málnási-Csizmadia, A., and Sellers, J.R. (2004). Mechanism of blebbistatin inhibition of myosin II. J. Biol. Chem. *279*, 35557–35563.

Kuure, S., Cebrian, C., Machingo, Q., Lu, B.C., Chi, X., Hyink, D., D'Agati, V., Gurniak, C., Witke, W., and Costantini, F. (2010). Actin depolymerizing factors cofilin1 and destrin are required for ureteric bud branching morphogenesis. PLoS Genet. *6*, e1001176.

Lappalainen, P., and Drubin, D.G. (1997). Cofilin promotes rapid actin filament turnover in vivo. Nature *388*, 78–82.

Li, M., Indra, A.K., Warot, X., Brocard, J., Messaddeq, N., Kato, S., Metzger, D., and Chambon, P. (2000). Skin abnormalities generated by temporally controlled RXRalpha mutations in mouse epidermis. Nature *407*, 633–636.

Lüke, Y., Zaim, H., Karakesisoglou, I., Jaeger, V.M., Sellin, L., Lu, W., Schneider, M., Neumann, S., Beijer, A., Munck, M., et al. (2008). Nesprin-2 Giant (NUANCE) maintains nuclear envelope architecture and composition in skin. J. Cell Sci. *121*, 1887–1898.

McLean, G.W., Brown, K., Arbuckle, M.I., Wyke, A.W., Pikkarainen, T., Ruoslahti, E., and Frame, M.C. (2001). Decreased focal adhesion kinase suppresses papilloma formation during experimental mouse skin carcinogenesis. Cancer Res. *61*, 8385–8389.

Miralles, F., Posern, G., Zaromytidou, A.I., and Treisman, R. (2003). Actin dynamics control SRF activity by regulation of its coactivator MAL. Cell *113*, 329–342.

Mizuno, K. (2013). Signaling mechanisms and functional roles of cofilin phosphorylation and dephosphorylation. Cell. Signal. *25*, 457–469.

Mouneimne, G., DesMarais, V., Sidani, M., Scemes, E., Wang, W., Song, X., Eddy, R., and Condeelis, J. (2006). Spatial and temporal control of cofilin activity is required for directional sensing during chemotaxis. Curr. Biol. *16*, 2193–2205.

Nolen, B.J., Tomasevic, N., Russell, A., Pierce, D.W., Jia, Z., McCormick, C.D., Hartman, J., Sakowicz, R., and Pollard, T.D. (2009). Characterization of two classes of small molecule inhibitors of Arp2/3 complex. Nature *460*, 1031–1034.

Olson, E.N., and Nordheim, A. (2010). Linking actin dynamics and gene transcription to drive cellular motile functions. Nat. Rev. Mol. Cell Biol. *11*, 353–365.

Owens, D.W., Brunton, V.G., Parkinson, E.K., and Frame, M.C. (2000). E-cadherin at the cell periphery is a determinant of keratinocyte differentiation in vitro. Biochem. Biophys. Res. Commun. *269*, 369–376.

Pollard, T.D., and Borisy, G.G. (2003). Cellular motility driven by assembly and disassembly of actin filaments. Cell *112*, 453–465.

Pollard, T.D., and Cooper, J.A. (2009). Actin, a central player in cell shape and movement. Science *326*, 1208–1212.

Quintanilla, M., Brown, K., Ramsden, M., and Balmain, A. (1986). Carcinogen-specific mutation and amplification of Ha-ras during mouse skin carcinogenesis. Nature *322*, 78–80.

Reed, K.R., Athineos, D., Meniel, V.S., Wilkins, J.A., Ridgway, R.A., Burke, Z.D., Muncan, V., Clarke, A.R., and Sansom, O.J. (2008). B-catenin deficiency, but not Myc deletion, suppresses the immediate phenotypes of APC loss in the liver. Proc. Natl. Acad. Sci. USA *105*, 18919–18923.

Rizvi, S.A., Neidt, E.M., Cui, J., Feiger, Z., Skau, C.T., Gardel, M.L., Kozmin, S.A., and Kovar, D.R. (2009). Identification and characterization of a small molecule inhibitor of formin-mediated actin assembly. Chem. Biol. *16*, 1158–1168.

Sotiropoulos, A., Gineitis, D., Copeland, J., and Treisman, R. (1999). Signal-regulated activation of serum response factor is mediated by changes in actin dynamics. Cell *98*, 159–169.

Starr, D.A., and Fridolfsson, H.N. (2010). Interactions between nuclei and the cytoskeleton are mediated by SUN-KASH nuclear-envelope bridges. Annu. Rev. Cell Dev. Biol. *26*, 421–444.

Stewart, C.L., Roux, K.J., and Burke, B. (2007). Blurring the boundary: the nuclear envelope extends its reach. Science *318*, 1408–1412.

Straight, A.F., Cheung, A., Limouze, J., Chen, I., Westwood, N.J., Sellers, J.R., and Mitchison, T.J. (2003). Dissecting temporal and spatial control of cytokinesis with a myosin II Inhibitor. Science *299*, 1743–1747.

Tahtamouni, L.H., Shaw, A.E., Hasan, M.H., Yasin, S.R., and Bamburg, J.R. (2013). Non-overlapping activities of ADF and cofilin-1 during the migration of metastatic breast tumor cells. BMC Cell Biol. *14*, 45.

Uehata, M., Ishizaki, T., Satoh, H., Ono, T., Kawahara, T., Morishita, T., Tamakawa, H., Yamagami, K., Inui, J., Maekawa, M., and Narumiya, S. (1997). Calcium sensitization of smooth muscle mediated by a Rho-associated protein kinase in hypertension. Nature *389*, 990–994.

Vartiainen, M.K., Mustonen, T., Mattila, P.K., Ojala, P.J., Thesleff, I., Partanen, J., and Lappalainen, P. (2002). The three mouse actin-depolymerizing factor/cofilins evolved to fulfill cell-type-specific requirements for actin dynamics. Mol. Biol. Cell *13*, 183–194.

Vartiainen, M.K., Guettler, S., Larijani, B., and Treisman, R. (2007). Nuclear actin regulates dynamic subcellular localization and activity of the SRF cofactor MAL. Science *316*, 1749–1752.

Versaevel, M., Braquenier, J.B., Riaz, M., Grevesse, T., Lantoine, J., and Gabriele, S. (2014). Super-resolution microscopy reveals LINC complex recruitment at nuclear indentation sites. Sci. Rep. *4*, 7362.

Wang, W., Eddy, R., and Condeelis, J. (2007). The cofilin pathway in breast cancer invasion and metastasis. Nat. Rev. Cancer *7*, 429–440.

Wiggan, O., Shaw, A.E., DeLuca, J.G., and Bamburg, J.R. (2012). ADF/cofilin regulates actomyosin assembly through competitive inhibition of myosin II binding to F-actin. Dev. Cell *22*, 530–543.

Worth, D.C., Hodivala-Dilke, K., Robinson, S.D., King, S.J., Morton, P.E., Gertler, F.B., Humphries, M.J., and Parsons, M. (2010). Alpha v beta3 integrin spatially regulates VASP and RIAM to control adhesion dynamics and migration. J. Cell Biol. *189*, 369–383.

Zhen, Y.Y., Libotte, T., Munck, M., Noegel, A.A., and Korenbaum, E. (2002). NUANCE, a giant protein connecting the nucleus and actin cytoskeleton. J. Cell Sci. *115*, 3207–3222.

SUPPLEMENTAL INFORMATION

SUPPLEMENTAL DATA

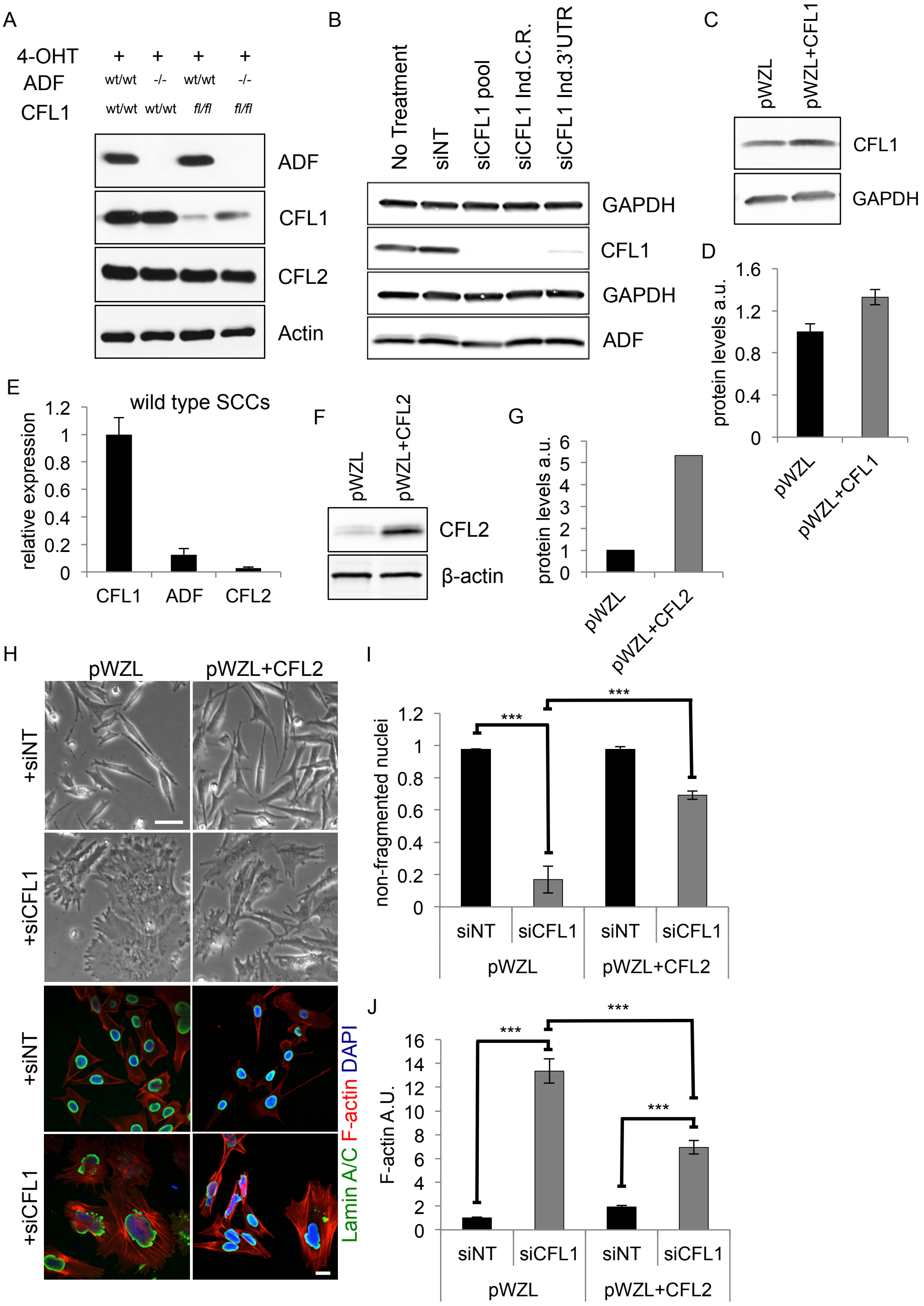

Figure S1. Depletion and re-expression of ADF/Cofilins (related to Figures 1, 2 and 5). (A) K14CreER^{T2} and K14CreER^{T2}/ADF$^{-/-}$/CFL1$^{wt/wt}$, K14CreER^{T2}/ADF$^{wt/wt}$/CFL1$^{fl/fl}$ and K14CreER^{T2}/ADF$^{-/-}$/CFL1$^{fl/fl}$ mice were treated with 4-OHT. Proteins

were harvested from mouse tails and immunoblotting performed using anti-ADF, anti-CFL1, anti-CFL2 and anti-actin antibodies. **(B)** siRNA knock-down efficiency for CFL1 was tested by immunoblotting at 72h post-transfection of 32nM siRNA in wild type (wt) Squamous Cell Carcinoma cells (wt-SCCs). A pool of 4 siRNAs targeting CFL1 (siCFL1 pool) as well as individual siRNAs targeting the coding region (siCFL1 Ind.C.R.) or the untranslated (siCFL1 Ind.3'UTR) region of CFL1-mRNA were utilized. siNT represents non-targeting control siRNAs. GAPDH was used as a loading control. **(C)** Western blot comparing CFL1 levels in ADF-null SCCs infected with retroviruses containing an empty vector (pWZL) or a wt CFL1 expressing vector (pWZL+CFL1). **(D)** Quantification of the immunoblots using Image J based on the intensity of the protein species and normalized to the loading control (n=2, ± S.D.). **(E)** Relative expression of ADF, CFL1 and CFL2 in wt SCCs. Results were generated from two individual cell lines and data is presented ± S.D. The amplification efficiency of the primers was determined and taken into account for the data analysis after the results were normalized to an endogenous control (GAPDH). The primers used are provided in Table S1. **(F)** Western blot comparing CFL2 levels in ADF-null SCCs infected with retroviruses containing an empty vector (pWZL) or a wt CFL2 expressing vector (pWZL+CFL2). **(G)** Quantification of the immunoblots in (F) performed as described in (D). **(H)** Phase contrast images of ADF-null SCCs infected with an empty (pWZL) or CFL2 expressing vector (pWZL+CFL2) and treated with siNT or siCFL1 (top panels). Cells were stained for F-actin (red) and DAPI (blue) (bottom panels). Scale bars 60μm for phase and 20μm for IF images, respectively. **(I)** Quantification of pWZL or pWZL+CFL2 containing cells that displayed non-fragmented nuclei after siRNA treatment. Results presented as mean ± SEM (n=4). >100 cells were counted per condition per repeat. *** One-way ANOVA with Tukey's multiple comparison test, p-value ≤ 0.0001. **(J)** Quantification of F-actin levels of the same cells as in (I). ≥69 cells quantified per condition (n=3). Data presented as mean ± SEM. *** One-way ANOVA with Tukey's multiple comparison test, p-value ≤ 0.0001.

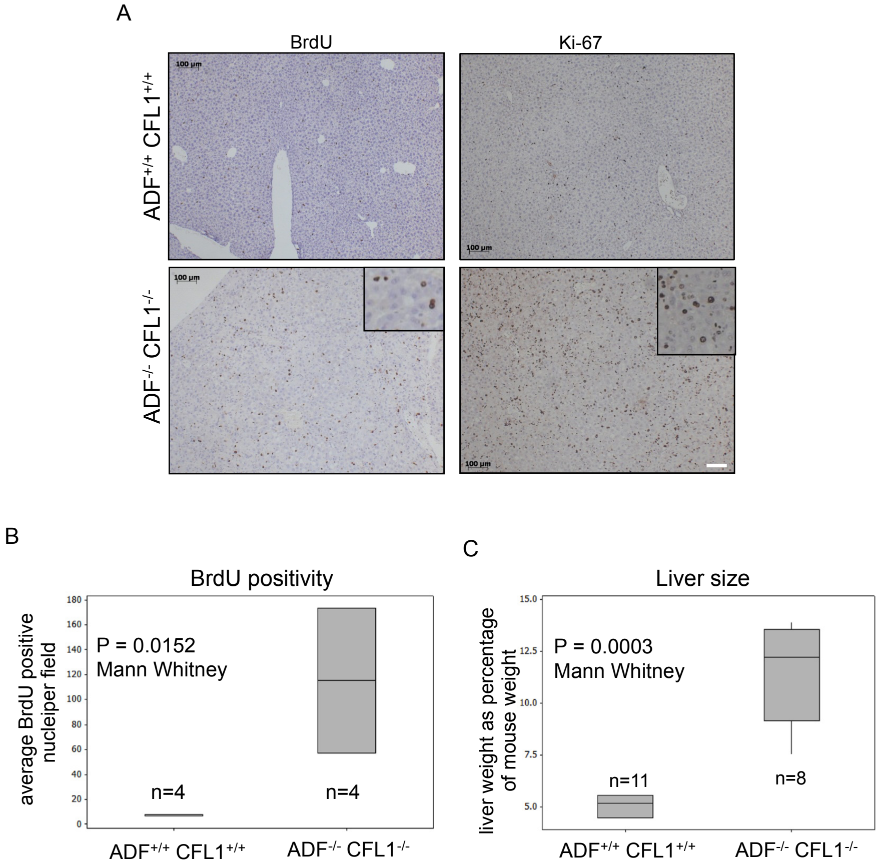

Figure S2. ADF and CFL1 gene deletion *in vivo* triggers hepatocyte hyperproliferation and liver enlargement (related to Figure 1). (A) As in skin, loss of ADF and CFL1 promotes liver hyperplasia. AhCreADF$^{-/-}$ CFL1$^{fl/fl}$ mice were treated with a single injection of β-naphthoflavone (80 mg/kg) to induce CFL1 gene deletion and aged for 8-9 days post induction. ADF$^{-/-}$ CFL1$^{-/-}$ livers show increased levels of proliferation as evidenced by greater BrdU incorporation or Ki67 staining by immunohistochemistry (IHC). Scale bar 100µm. **(B)** Quantification of BrdU positive nuclei is shown. At least 3 fields per liver were scored. **(C)** Liver weights were measured (g) on dissection and are presented as percent total body weight. The ADF$^{-/-}$ CFL1$^{-/-}$ livers show significant hepatomegaly compared to controls, confirming the enlarged, hardened appearance of the liver on dissection.

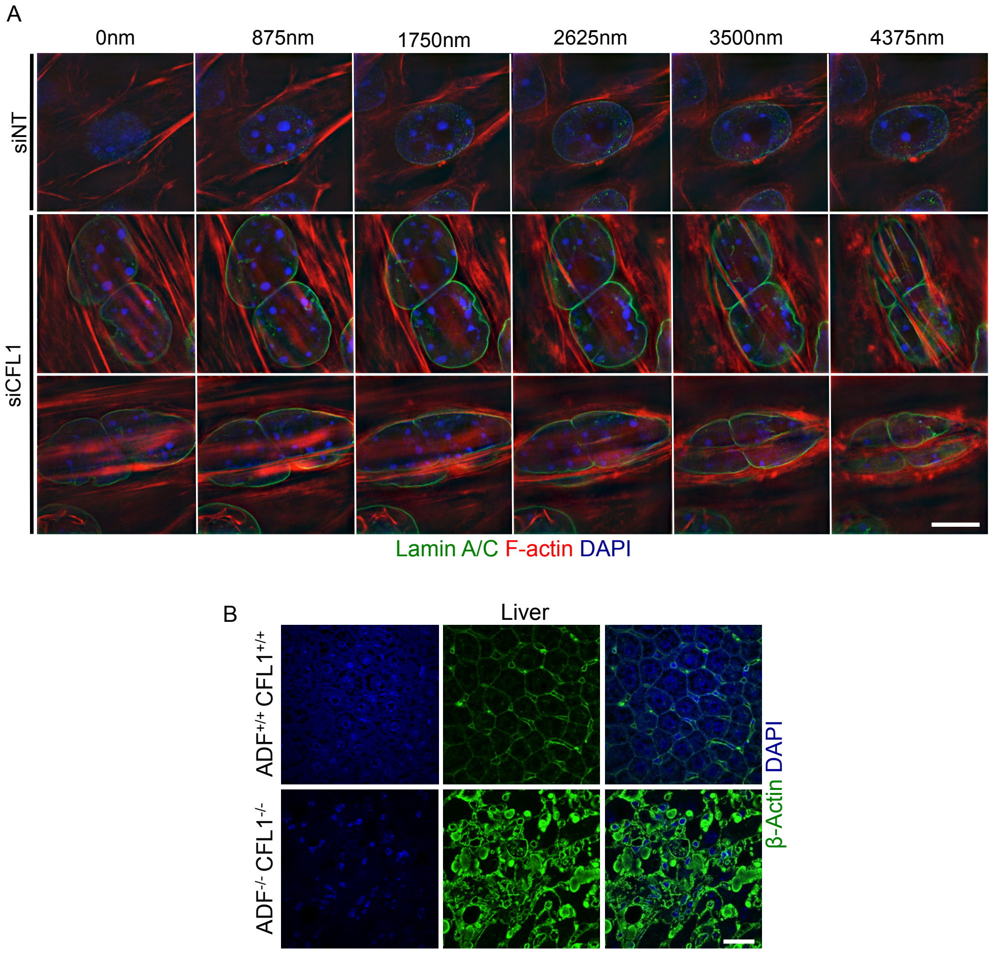

Figure S3. Structured Illumination Microscopy (SIM) and β-actin staining in liver (related to Figure 4). (A) High resolution z-stack of cells treated for 48h with siRNAs for CFL1 or non-targeting, obtained by SIM imaging. Cells stained for Lamin A/C (green), F-actin (red) and DAPI (blue). Scale bar 10μm. **(B)** AhCreADF$^{-/-}$ CFL1$^{fl/fl}$ mice were treated with a single injection of β-naphthoflavone (80 mg/kg) to induce CFL1 gene deletion and aged for 8-9 days post induction. ADF$^{-/-}$ CFL1$^{-/-}$ livers show increased F-actin when stained for β-actin via immunofluorescence (IF). Scale bar 20μm.

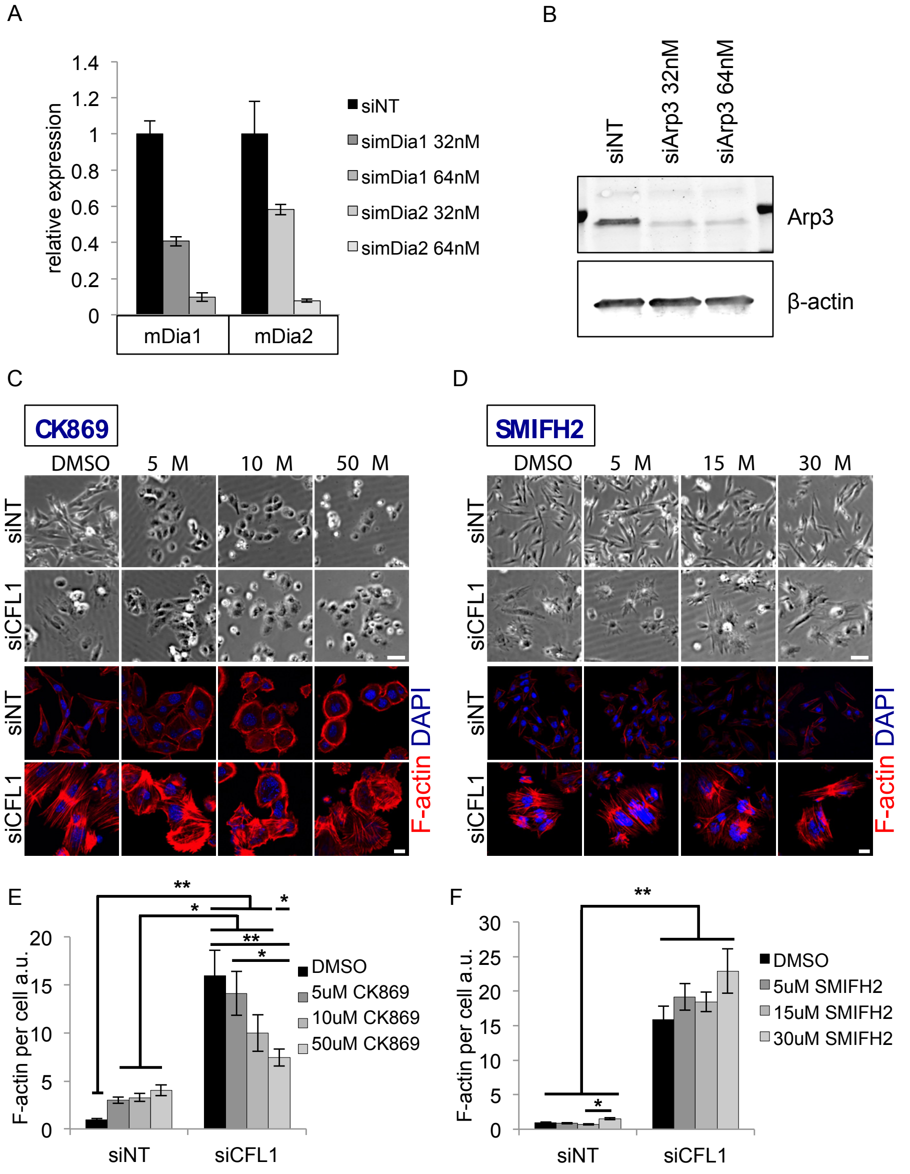

Figure S4. An inhibitor of Arp3, but not formins, suppresses F-actin accumulation following ADF/CFL1 depletion (related to Figure 5). (A) Quantitative PCR was performed to show knock-down of protein expression in cells treated for 72h with 32nM or 64nM of siRNA pools targeting mDia1 and mDia2. The data was normalized against cells treated with 64nM siNT. Bars represent ± S.D. The primers are shown in Table S1. **(B)** Immunoblot for siRNA-mediated knock-down of Arp3. Cells were treated for 72h with two different concentrations of siRNA pools (32nM and 64nM) targeting Arp3. β-actin used as a loading control. **(C-D)** *Upper panels*: Phase contrast images of ADF-null SCCs treated for 30h with siRNAs for siCFL1 or non-targeting siNT, and subsequently incubated overnight with the addition of DMSO or increasing concentrations of inhibitors for Arp2/3 (CK869) or

formins (SMIFH2). *Lower panels*: Cells were stained for F-actin (red) and DAPI (blue). Scale bars 60μm for phase and 25μm for IF images, respectively. **(E-F)** Quantification of F-actin levels per cell was performed using IF confocal images. Cells were stained with phalloidin and measurements were carried out in Image J. ≥18 cells contained in ≥4 fields were quantified per treatment. One representative experiment of two performed is shown. Data presented as mean ± SEM. * One-way ANOVA, p-value ≤ 0.05, ** p-value ≤ 0.002.

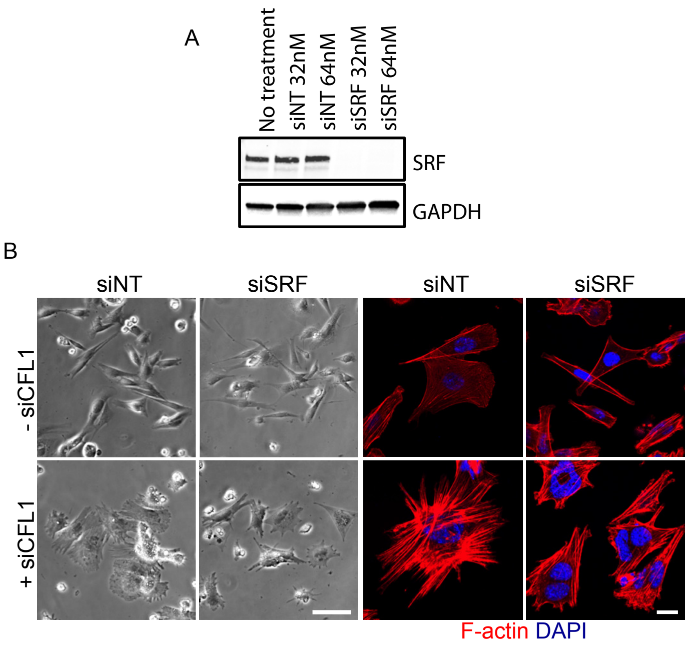

Figure S5. SRF depletion in ADF-null SCC cells (related to Figure 5). (A) Knock-down efficiency of siRNAs targeting SRF was tested by immunoblotting. siRNAs were used in two concentrations, 32nM and 64nM. GAPDH was used as a loading control. **(B)** Phase contrast images of ADF-null SCC cells treated with siNT and siCFL1 alone or alongside siRNAs targeting SRF (siSRF) (left panels). Cells were stained for F-actin (red) and DAPI (blue) (right panels). Scale bars 125μm for phase and 20μm for IF images, respectively.

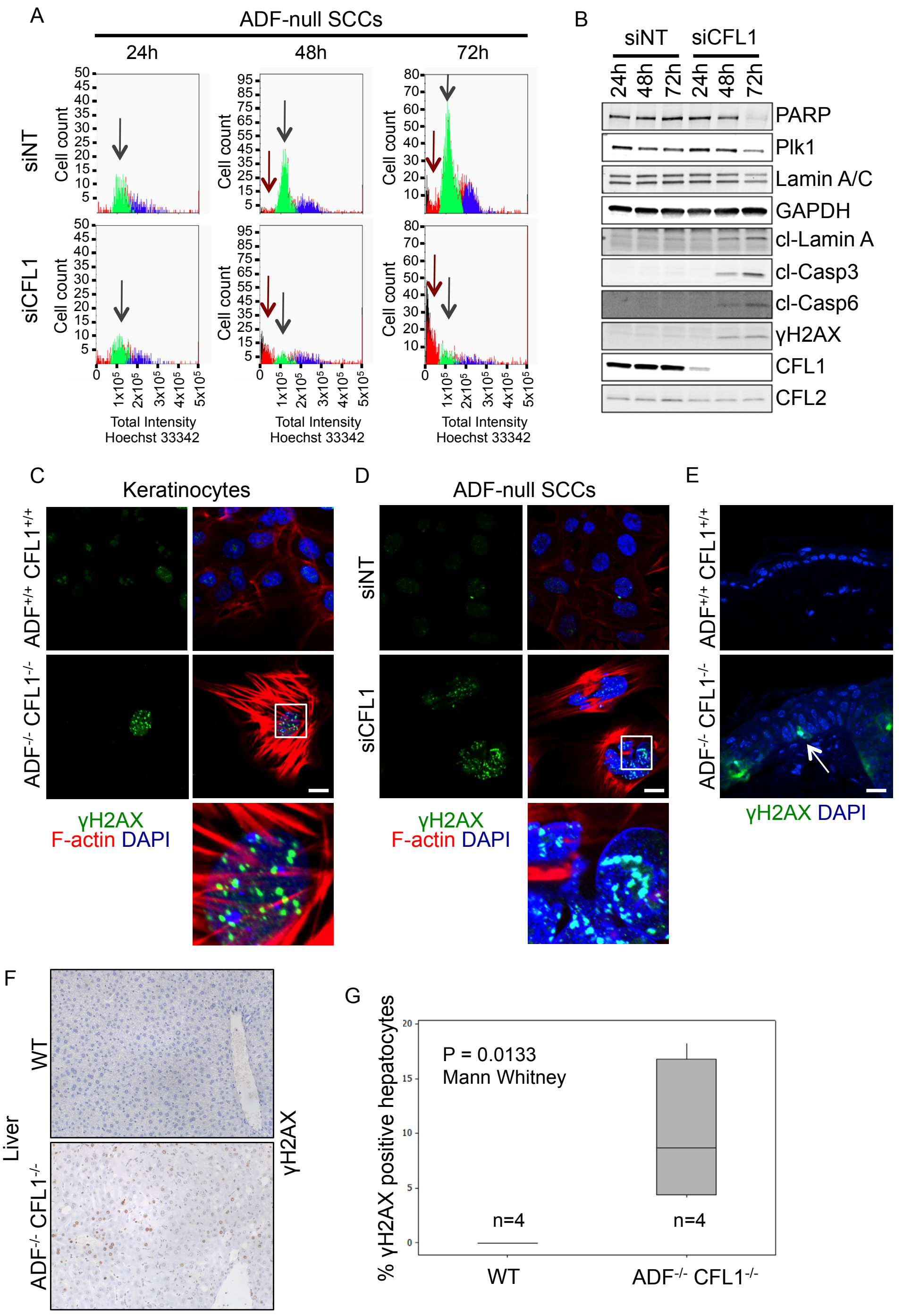

Figure S6. ADF/CFL1 depletion promotes DNA damage and damage-induced signaling (related to Figure 7). (A) Cell cycle profile over 72h of ADF-null SCC cells treated with either siNT or siCFL1. Hoechst 33342 stained nuclei were imaged and analyzed using the Olympus Scan^R acquisition software. Nuclei were gated according to their total intensity, which correlates with total DNA content and subsequently the stage of the cell cycle. Black arrows indicate G1 phase and red arrows show a sub-G1 shift of siRNA treated cells. Experiment repeated twice with

two different cell lines. Pooled data of two cell lines from one representative experiment is shown. **(B)** Immunoblots showing damage-induced apoptotic markers in ADF-null SCC cells treated for 24h, 48h and 72h with siRNAs for CFL1 or non-targeting controls. **(C)** Primary tail keratinocytes isolated from $ADF^{+/+}$ $CFL1^{+/+}$ and $ADF^{-/-}$ $CFL1^{-/-}$ mice were stained with anti-γH2AX (green), TRITC-phalloidin (red) and DAPI (blue). Zoomed image below panel. Scale bar 20μm. **(D)** ADF-null SCCs treated with siNT and siCFL1 for 48h. Cells stained as in (C); zoomed image is provided below panel. Scale bar 20μm. **(E)** Immunofluorescence staining of paraffin-embedded skin sections from $ADF^{+/+}$ $CFL1^{+/+}$ and $ADF^{-/-}$ $CFL1^{-/-}$ mice. Sections stained with anti-γH2AX (green) and DAPI (blue). White arrow indicates DNA-damage associated positive staining in the double knock-out section. Scale bar 20μm. **(F-G)** AhCre$ADF^{-/-}$ $CFL1^{fl/fl}$ mice were treated with a single injection of β-naphthoflavone (80 mg/kg) to induce CFL1 gene deletion and aged for 8-9 days post induction. $ADF^{-/-}$ $CFL1^{-/-}$ livers showed significantly increased levels of γH2AX when stained by IHC. 4 mice were stained for each genotype and at least 3 fields per liver.

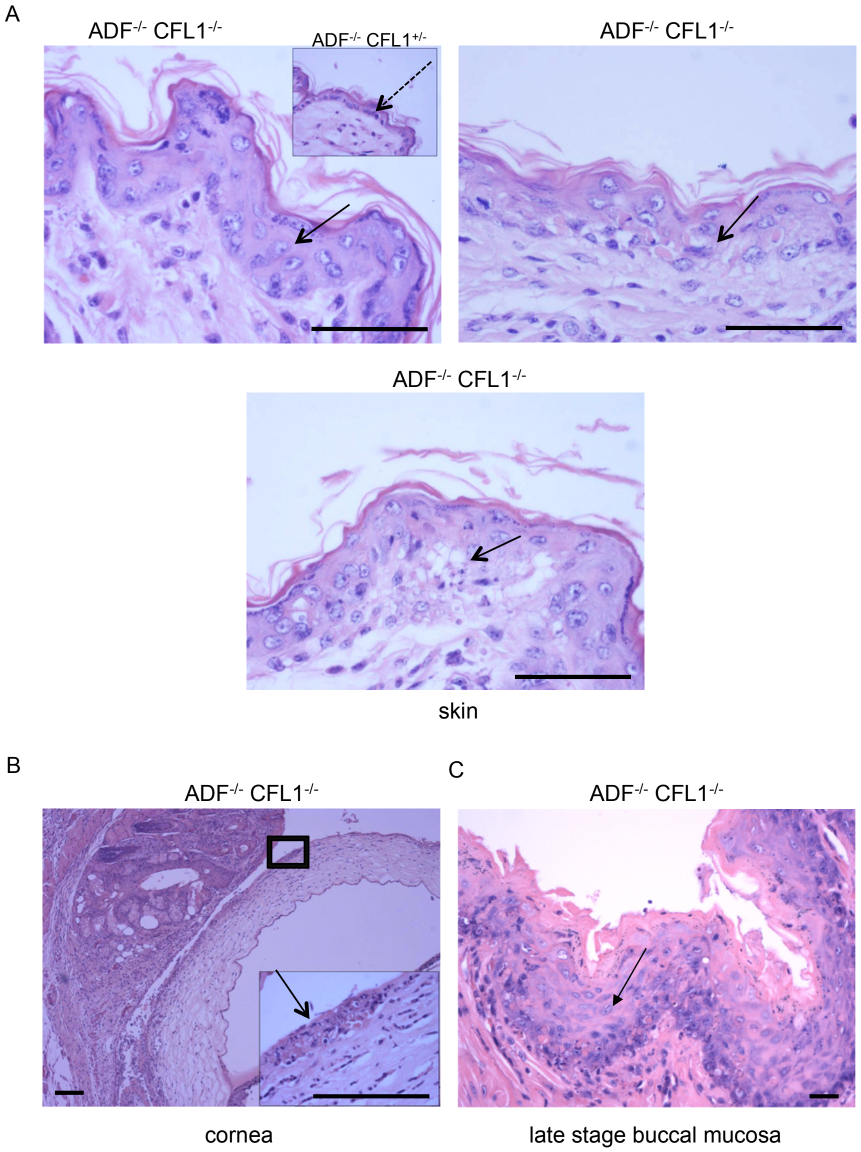

Figure S7. Deletion of ADF and CFL1 causes pathological loss of tissue homeostasis (related to Figure 1 and throughout). (A) Paraffin-embedded skin sections from K14CreER^{T2}/ADF$^{-/-}$/CFL1$^{wt/fl}$ (ADF$^{-/-}$ CFL1$^{+/-}$) (upper left panel, inset) and K14CreER^{T2}/ADF$^{-/-}$/CFL1$^{fl/fl}$ (ADF$^{-/-}$ CFL1$^{-/-}$) mice treated with 4-OHT were stained with hematoxylin and eosin (H & E). Solid arrow in upper left panel indicates presence of hypertrophic epidermis with jumbled keratinocytes while broken arrow shows the comparison with a normal epidermal layer. Solid arrow in upper right panel indicates apoptotic and vacuolated cells within the epidermis while arrow in lower panel shows formation of early vesicles. **(B)** Paraffin-embedded sections of murine cornea from ADF$^{-/-}$ CFL1$^{-/-}$ mice were stained with H & E. Solid arrow in inset zoomed image shows ulcerated corneal epithelium. **(C)** Paraffin-embedded sections of murine buccal mucosa from ADF$^{-/-}$ CFL1$^{-/-}$ mice were stained with H & E. Solid

arrow indicates ulcerative stomatitis. Scale bars 100μm.

Movie S1. ADF/CFL1 depleted cells are capable of membrane protrusion (related to Figure 2E). ADF-null SCCs were treated with non-targeting (siNT) siRNAs. After 48h the cells were imaged every 30 seconds for 30 minutes. This movie provides control for Movie S2.

Movie S2. ADF/CFL1 depleted cells are capable of membrane protrusion (related to Figure 2E). ADF-null SCCs were treated with CFL1 (siCFL1) siRNAs. After 48h the cells were imaged every 30 seconds for 30 minutes. Control transfected with non-targeting (siNT) siRNAs is presented in Movie S1.

Table S1 (Related to Figures 7B, S1E and S4A). List of primers used for quantitative PCR experiments.

target	primer	sequence
Nesprin-2G	forward	tagacgcggacatgcaac
	reverse	ctcaatctctgtcagcatagcc
mDia1	forward	ggtacccgggacaagaaga
	reverse	ctcatgctggtaaatctctcca
mDia2	forward	cctaaaattgctcagaacctttca
	reverse	aatatcatcgtcctgattttctca
ADF	forward	gctttgagaccaaggagtcca
	reverse	tcagaggtgcttgttctggtg
CFL1	forward	tctgtctccctttcgtttcc
	reverse	cattgaacaccttgatgacacc
CFL2	forward	acgtacgaaacaaaagagtctaagaa
	reverse	gcataaatcatcttgctttttaacg
GAPDH	forward	atggtgaaggtcggtgtga
	reverse	aatctccactttgccactgc

SUPPLEMENTAL EXPERIMENTAL PROCEDURES

Generation of transgenic mice

Design, construction, and generation of transgenic mice with either deletion of ADF (Gurniak et al., 2005), targeted loxP sites in the *CFL1* gene (Bellenchi et al., 2007) or expressing the modified Cre recombinase-estrogen receptor fusion under control of the Keratin-14 promoter (K14CreER^{T2}; (Li et al., 2000)) have been described. To enable cell-type-specific CFL1 ablation ADF$^{-/-}$ mice and CFL1$^{fl/fl}$ mice were mated to K14CreER^{T2} transgenic mice. This resulted in offspring that carried K14CreER^{T2} and were nullizygous for ADF and homozygous for the floxed *CFL1* gene (K14CreER^{T2}/ADF$^{-/-}$/CFL1$^{fl/fl}$) or offspring that were nullizygous for ADF but carried two copies of the wild-type *CFL1* allele (K14CreER^{T2}/ADF$^{-/-}$/CFL1$^{wt/wt}$) or offspring that carried two copies of the wild-type *ADF* allele but were homozygous for the floxed *CFL1* gene (K14CreER^{T2}/ADF$^{wt/wt}$/CFL1$^{fl/fl}$). Genotypes were confirmed by PCR analysis. All animals used were crossed onto and maintained on an FVB genetic background. All experiments were carried out in accordance with the UK Coordinating Committee on Cancer Research guidelines by approved protocol (Home Office Project License no. 60/4248).

Preparation and administration of 4-OHT

Tamoxifen (4-OHT) was prepared and administered to 60-day-old K14-ADF mice as described (Indra et al., 1999) except that a reduced dose of 100μg in 100μl of sunflower oil was used. The animals were then left for 10 days before experiments initiated except for K14CreER^{T2}/ ADF$^{-/-}$ CFL1$^{fl/fl}$ mice, which were culled and tissue collected at 4-5 days. 4-OHT for *in vitro* experiments was dissolved in ethanol and used at a final concentration of 10nM for 24-48 hours.

Generation of Squamous Cell Carcinoma cell lines

Ras-driven chemically induced tumours were introduced to mice as previously described (McLean et al., 2001). Squamous Cell Carcinoma (SCC) tumours derived from mice nullizygous to ADF were then removed, cut into small pieces and trypsinised (0.25% in PBS-EDTA, Gibco) for 10min at RT. Trypsin was neutralized with the addition of DMEM + 10% FBS in the presence of 100 Units penicillin and 100μg streptomycin per ml (Sigma). Antibiotics were removed after five passages and

the ADF-null Squamous Cell Carcinoma cells (SCCs) were considered a cell line once reached a stable growing capacity. SCCs were cultured in Dulbecco's Modified Eagle Medium (Sigma) supplemented with 10% FBS (Gibco). All cell cultures were maintained in 37°C, 5% CO_2.

Isolation of primary keratinocytes from mouse tails

Tails were taken from the mice, cut lengthwise and the skin removed. Skins were washed in sterile PBS prior to overnight incubation at 4°C or 2h incubation at 37°C in Dispase (4 mg/ml in PBS, Gibco). The epidermis was removed, cut into several pieces, trypsinized (0.25% in PE, Gibco) for 10min at 37°C and vortexed to dissociate the cells. Cells were re-suspended in Dulbecco's modified Eagle's medium (DMEM, Sigma) + 20% FBS (Gibco) and passed through a 70µm cell strainer (Becton Dickinson). Samples were centrifuged at 1100 rpm for 5 minutes, washed with PBS, centrifuged again and the pellet re-suspended in Keratinocyte Basal Medium (KBM) + KGM singlequotes (Clonetics). Cells were then plated on collagen I-coated cellware (Becton Dickinson) or lysed as described.

CFL1 cloning and overexpression

CFL1 cDNA was cloned into the pWZL-Hygro retroviral vector with a standard DNA ligation kit protocol (Roche). pEco packaging cells were used for viral production and subsequently SCC cells were infected with addition of polybrene (Millipore). Successfully transformed cells were selected using 1:100 Hygromycin (Calbiochem).

Western blotting

Cells were lysed directly in RIPA [50mM Tris–HCl, pH 7.4, 150mM NaCl, 0.1% SDS, 1% NP-40 and 0.5% deoxycholate] with inhibitors (cOmplete ULTRA and phosSTOP tablets, Roche). Clarification was by high-speed centrifugation (13,000 rpm at 4°C for 15min). 20µg of lysates were separated by SDS-PAGE, transferred to nitrocellulose and immunoblotted. Antibodies used were as follows: anti-Destrin (Pierce), anti-Cofilin-1 (CST), anti-Cofilin-2 (m-Cofilin KG-60 from W. Witke or Abcam), anti-Actin/GAPDH/PARP/Myosin-IIA/Lamins/Caspases and γH2AX (all CST), anti-Plk-1 (Millipore) and anti-SUN1 (Santa Cruz). The majority of western blots were analysed using LI-COR Odyssey infrared imaging system and Image Studio software (version 4.0.21). Signal was detected using 680 and 800 IRDye fluorescent antibodies (LI-COR). The software settings were chosen to maximise

signal-to-noise ratio where saturation was not reached and color images generated were converted to grayscale. The blots for β–actin, GAPDH, CFL1 and Plk1 had very low/undetectable background. Western blots presented in Figures 3A and S1A were analysed using traditional luminescence-based detection system.

Immunohistochemistry and BrdU labeling and quantification

Dorsal skin samples were harvested from mice and fixed in neutral-buffered formaldehyde 10% vol/vol (Surgipath Europe), embedded in paraffin and cut into five micron sections for H and E staining or immunohistochemistry. Sections were deparaffinized, rehydrated and unmasked in 10mM citric acid (pH 6.0) in a pressure cooker then stained using DakoCytomation Envision kit appropriate to the species of primary antibody (DakoCytomation Ltd.). The following antibody dilutions were used: anti-Cytokeratin 6 1:1500 and anti-Cleaved Caspase-3 (Asp175) 1:200. All were incubated overnight at 4°C. Sections were counterstained with hematoxylin.

For BrdU labeling mice were injected (ip) with 0.2ml/20g of body weight BrdU (GE Healthcare) 2h prior to harvesting. Skin sections from labeled mice were stained with anti-BrdU antibody (1:1500, CST) as above. Quantification of BrdU positive nuclei from the mouse epidermis was performed with ImmunoRatio, a web-based application for image analysis (Tuominen et al., 2010).

Immunofluorescence

Sections were deparaffinized, rehydrated and unmasked in 10mM citric acid (pH 6.0) in a pressure cooker for 10 minutes. They were then blocked for 1 hour with 1% BSA/2% FBS in water (Blocking buffer). Primary antibodies diluted in Blocking buffer were incubated for 1 hour, washed 3 times for 5min in 0.1% Tween 20/TBS (TBST) and Alexa Fluor-488- or Alexa Fluor-594-conjugated secondary antibodies (Invitrogen) added for 1 hour RT. Sections rinsed 3 times for 5min in TBST and mounted with Vectashield Mounting Media containing DAPI (Vector Laboratories). For β-actin and Lamin A/C staining dorsal skin samples were harvested and fixed for 30 minutes at 37°C in 4% paraformaldehyde, cooled down to RT and incubated overnight at 4°C before being embedded in paraffin. 5μm sections were deparaffinized, rehydrated and unmasked as described above before being blocked with peroxidase and protein blocking buffers for 2h at RT (DakoCytomation) and incubated overnight at 4°C with primary antibodies.

The following antibody dilutions were used: anti-E-cadherin 1:100 (CST), anti-Cytokeratin 14 1:100 (made in-house), anti-Cytokeratin 10 1:1500, anti-Cytokeratin 6 1:1500 (Abcam), anti-β-actin and Lamin A/C 1:200 (CST).

Keratinocytes and SCCs were grown on glass coverslips, fixed and permeabilized at room temperature for 10 min (3.7% formaldehyde, 0.2% Triton X-100, 1mM $MgCl_2$, 100mM PIPES and 10mM EGTA), blocked for 1 hour (2% BSA in 0.1% Triton X-100/TBS) and incubated with primary antibodies overnight at 4°C. Antibody detection was via Alexa Fluor-488 or Alexa Fluor-594-conjugated secondary antibodies for 45 min at RT. Between steps, cells were washed with 0.1% Triton X-100/TBS.

The following antibody dilutions were used: anti-E-cadherin 1:100, Lamin A/C and Myosin-IIA 1:50, pMLC2 (Ser19) 1:200, p-paxillin 1:50, α-actinin 1:50, γH2AX 1:400 (all CST) and anti-Vinculin 1:100 (Abcam). F-actin was visualized using TRITC-Phalloidin (Sigma) or Rhodamine Phalloidin (Life Technologies) along with the secondary antibody mix.

All immunofluorescent experiments were visualized using an Olympus FV1000 confocal microscope equipped with an UPLANSAPO 60X/1.35 oil immersion objective. Super-resolution imaging was performed with Nikon's N-SIM super-resolution microscope equipped with 100x oil objective lens (N.A. 1.49).

RNA interference and protein co-depletions

siRNA reverse transfections were carried out using RNAiMAX lipofectamine reagent (Life Technologies) according to the company's standard protocol. Non-targeting and CFL1 targeting siRNAs (Dharmacon) were used at a final concentration 32nM. CFL1 knock-down experiments carried out for 48h. siRNAs for Arp3, mDia1, mDia2, SRF and Nesprin-2G (Dharmacon) were used at final concentration 64nM.

For all co-depletion experiments cells were treated with siRNAs targeting the proteins to be depleted alongside CFL1 at day 1. 24h later media was changed and cells were treated for further 48h with a mixture of siRNAs targeting CFL1 and the co-depleted protein. Control cells were treated either with non-targeting siRNAs alone or alongside siRNAs for the co-depleted proteins.

Live cell imaging, kymograph and membrane dynamics analysis

48h post siRNA treatment cells were imaged every 30 seconds for 30 minutes using a Scan^R high content screening station with a 20X phase objective (Olympus). Kymograph and membrane analysis were performed as previously described (Benjamin et al., 2010). Kymograph profiles were compiled using normalized pixel intensities from 1-pixel wide kymographs. Protrusion length was calculated based on the kymograph peaks and protrusion persistence was calculated from the peaks' width, representing the time for a full membrane extension and retraction.

Inhibitor treatments

Prior to treatment with inhibitors cells were transfected with siRNAs for CFL1 or non-targeting. 30h post transfection media was changed with fresh containing the inhibitors and cells were incubated overnight prior to fixation.

Free barbed–ends assay and ImageJ quantification

Free barbed-end formation was assessed and quantified by an adaptation of the method described previously (Worth et al., 2010). Cells plated on coverslips were permeabilized using 0.125 mg/ml saponin in the presence of 9μg biotin conjugated G-actin (Cytoskeleton). They were incubated for 2min in 37°C and subsequently fixed with the standard formaldehyde fixation protocol described above. Detection of biotinylated actin was performed using FITC-streptavidin (20μg/ml, Vector) and F-actin was labelled with TRITC-phalloidin (Sigma). Images were acquired with an Olympus FV-1000 confocal microscope equipped with an UPLANSAPO 60X/1.35 oil immersion objective. The laser and acquisition settings were kept the same for imaging every condition, ensuring that there was no pixel saturation. Quantification of total pixel intensity per cell was performed using ImageJ after background subtraction for each field individually. Same method was used for the quantification of F-actin.

G- and F-actin assay

For G-actin isolation 60mm dishes of cells treated with siRNAs were rinsed twice with CSK buffer (10mM PIPES pH 6.8, 50mM NaCl, 3mM $MgCl_2$, 300mM Sucrose). Then, 500μl of lysis buffer 1 were added (CSK buffer + 1% Triton X-100, 1μg/ml phalloidin, protein inhibitors (cOmplete and phosSTOP tablets (Roche))), and plates incubated for 5min at RT on shaker. Supernatants were then collected and SDS was added to a final concentration of 2%.

F-actin was isolated after washing the dishes three times with CSK buffer and

incubating 5min at RT on shaker with 500µl of lysis buffer 2 (Lysis Buffer 1 + 2% SDS). The cells were then scraped and the samples were homogenized with syringes and 25 gauge needles. Protein assay was done on G-actin sample and equivalent amounts of lysates were run on a 4-12% polyacrylamide gel.

Cell cycle analysis

Cell cycle analysis was image based using the Scan^R high content screening station (Olympus). Cells were classified in G1 or S/G2/M phases according to their nuclear intensity which depends on the nuclear content. Nuclei were stained for 1h at 37°C with Hoechst 33342 (Invitrogen) prior to imaging. Image analysis was performed with the ScanR Image Analysis software.

Deletion of genes encoding ADF and CFL1 in mouse liver

AhCre^{+} mice (Ireland et al., 2004) were intercrossed with ADF$^{-/-}$/CFL1$^{fl/fl}$ mice. To induce recombination mice were given a single injection of 80mg/kg ß-naphthoflavone (IP). For BrdU labeling, mice were injected 250µl BrdU (GE Healthcare) 2h prior to harvesting. Staining was performed using an anti-BrdU antibody (BD Bioscience) at 1:200 or Ki-67 (Thermo) at 1:100 on formalin fixed liver sections.

Quantitative RT-PCR

RNA was extracted from cells using the RNeasy kit (QIAGEN) and then converted to cDNA with SuperScript First-Strand cDNA synthesis kit with Random Hexamer primers (Invitrogen). qPCR reactions were prepared with the SensiFAST Hi-ROX kit (Bioline) and run on StepOnePlus Real Time PCR system (Life Technologies). All procedures were done according to manufactures standard protocols. Results were analyzed with ΔΔCt method using GAPDH as endogenous control, and statistical significance was assessed with Student's t-test. All primers were designed in Universal ProbeLibrary Assay Design Center (Roche) and can be found in **Table S1**.

Statistical analysis

Statistical analysis was performed in Prism or in Excel for unpaired t-tests. Normality of the data of every experiment was assessed with D'Agostino & Pearson's normality test. When data was fitted in a normal distribution, statistical significance was assessed by Student's t-test in case of two groups, or by One-way ANOVA and

Tukey's multiple comparisons test for bigger groups. If data was not normally distributed, then Mann-Whitney's or Kruskal–Wallis one-way analysis of variance accompanied by Dunn's multiple comparisons test were utilised to ascertain statistical significance between two or more groups accordingly. Statistical significance was assumed for p-values < 0.05. Sample sizes, replicates and test used are indicated for each experiment in the figure legends.

SUPPLEMENTAL REFERENCES

Ireland, H., Kemp, R., Houghton, C., Howard, L., Clarke, A.R., Sansom, O.J., and Winton, D.J. (2004). Inducible Cre-mediated control of gene expression in the murine gastrointestinal tract: effect of loss of beta-catenin. Gastroenterology *126*, 1236-1246.

McLean, G.W., Brown, K., Arbuckle, M.I., Wyke, A.W., Pikkarainen, T., Ruoslahti, E., and Frame, M.C. (2001). Decreased focal adhesion kinase suppresses papilloma formation during experimental mouse skin carcinogenesis. Cancer Res. *61*, 8385-8389.

Tuominen, V.J., Ruotoistenmaki, S., Viitanen, A., Jumppanen, M., and Isola, J. (2010). ImmunoRatio: a publicly available web application for quantitative image analysis of estrogen receptor (ER), progesterone receptor (PR), and Ki-67. Breast Cancer Res. *12*.

Worth, D.C., Hodivala-Dilke, K., Robinson, S.D., King, S.J., Morton, P.E., Gertler, F.B., Humphries, M.J., and Parsons, M. (2010). alpha nu beta 3 integrin spatially regulates VASP and RIAM to control adhesion dynamics and migration. J. Cell Biol. *189*, 369-383.
